# V‐Shaped Heterostructure Nanocavities Array with CM and EM Coupled Enhancement for Ultra‐Sensitive SERS Substrate

**DOI:** 10.1002/advs.202409838

**Published:** 2024-10-28

**Authors:** Abdur Rahim, Liqi Ma, Muhammad Saleem, Baiju Lyu, Muhammad Shafi, Yuxin You, Mingyue Li, Xiaoyu Zhang, Mei Liu

**Affiliations:** ^1^ School of Physics and Electronics Shandong Normal University Jinan 250038 China

**Keywords:** AAO templates, organic/inorganic heterostructure, photodegradation, stability, surface‐enhanced Raman scattering

## Abstract

The field of semiconductor surface‐enhanced Raman scattering (SERS) substrates has experienced significant advancements, leading to a wide range of applications in several fields. However, the quest for new ultra‐sensitive semiconductor SERS materials is still of utmost importance. In this regard, an efficient and novel substrate, F_4_TCNQ/MoS_2_ heterostructure is introduced, assisted by V‐shaped aluminum anodic oxide (AAO) nanocavities with different depths. Utilizing the efficient charge transfer of organic/inorganic semiconducting heterostructure and the photoconfinement capability of the nanocavity structure of the AAO nanotemplate, excellent stability, fast sensing, enhanced Raman, and photodegradation activities are achieved. Due to its unique 3D structure, the optimized F_4_TCNQ/MoS_2_/AAO with 1500 nm depth achieves ultra‐high sensitivity detection of 9.0×10^−16^ M for conventional probe molecules. Furthermore, precise detection of water contaminants is observed for the first time with a V‐shaped heterostructure due to combined organic/inorganic features that differ significantly from conventional MoS_2_ structures or other metal/inorganic or inorganic/inorganic semiconductors. This research presents a novel and versatile strategy for SERS and demonstrates its diverse potential performance in practical applications.

## Graphical abstract

A novel V‐shaped F_4_TCNQ/MoS_2_ SERS substrate is demonstrated to exhibit ultra‐sensitive SERS performance for effective trace detection of water pollution with heavy metal ions (Hg^2+^) and potential photodegradation activity for pollutants. The composite substrate combines electromagnetic, chemical, and nanocavities structure enhancement, providing an ultra‐sensitive non‐metal detection platform for environmental analysis.

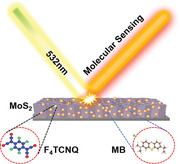

## 1 Introduction

Surface‐enhanced Raman scattering (SERS) is an emerging technique in various research fields due to its fast, sensitive, and nondestructive merits for detecting very small amount of molecules or even single molecule.^[^ ^1^, ^2^, ^3^, ^4^ ^]^ This technique was made possible by utilizing specially designed substrates that can enhance the Raman signal up to 10^14^ times.^[^ ^5^ ^]^ This tremendous enhancement is attributed to two dominant mechanisms: the electromagnetic mechanism (EM) and the chemical mechanism (CM).^[^ ^6^, ^7^ ^]^ The EM originates from localized surface plasmon resonances close to the noble metal surface (Au and Ag) by irradiating laser light. However, its high cost, easy agglomeration, nonreusability, poor biocompatibility, and low chemical stability hinder its applications in material and biological science.^[^ ^8^ ^]^ The CM‐based SERS effect mainly arises from charge transfer (CT) occurring between substrate and adsorbed probe molecules. In recent years, 2D semiconducting materials have drawn significant attention from the SERS research community owing to their excellent stability, reproducibility, layer‐dependent optical properties, high surface area, low cost, and superior biocompatibility attributed to CM.^[^ ^9^ ^]^ Among 2D materials, MoS_2_ has been extensively studied due to its unique electronic, optical, and mechanical properties applicable to SERS.^[^ ^10^ ^]^ Furthermore, organic semiconductors possess potential applications due to delocalized pi‐electrons, low‐cost production, flexibility, additional charge transfer, and enhanced affinity.^[^ ^11^ ^]^ However, compared to inorganic 2D semiconductors organic materials suffer from low mobility, thermodynamic instability, and growth disorders. To overcome such limitations, constructing organic/inorganic hybrid heterostructures is a good approach by utilizing both the advantages of small organic and inorganic 2D semiconductors.^[^ ^12^ ^]^ The organic/inorganic heterojunction improves low mobility by forming delocalized molecular orbitals or band gap structures, which ensures efficient charge transport under the influence of an electric field. This results in high carrier mobility (i.e., electron or hole) and low effective carrier mass.^[^ ^13^ ^]^ When molecular systems are in close proximity to these semiconductors, the presence of charge‐carrier electrons in the high‐energy occupied molecular orbitals (or valence band edge) and empty orbital states in the low‐energy unoccupied molecular orbitals (or conduction band edge) provides numerous pathways for charge/energy‐transfer processes within the semiconductor‐molecule system under the influence of an electric field or electromagnetic radiation. These charge transfers contribute significantly to the SERS mechanism.^[^ ^12^, ^13^ ^]^ To overcome the inferior thermodynamic stability, the inorganic material (MoS_2_) possesses excellent thermal conductivity, which helps in dissipating the heat generated during SERS measurements. This prevents the localized overheating that can cause structural damage to the organic molecules. Studies have shown that integrating materials with high thermal conductivity can significantly enhance the overall thermal stability of the composite substrate.^[^ ^14^ ^]^

Designing organic/inorganic hybrid materials makes composites less thermally conductive (more thermally stable) and more flexible. e,g., using organic thin films combined with black phosphorene and MoS_2_ heterojunction with graphene proved long‐term stability, highlighting the potential of heterojunctions to significantly increase the stability of long‐term SERS substrates.^[^ ^14^, ^15^, ^16^ ^]^ Organic semiconductors often experience growth disorder, which can be mitigated by using 2D inorganic materials as template substrates. These inorganic materials, with their ultra‐flat, dangling bond‐free surfaces and high carrier mobility, facilitate the high‐quality growth of organic materials with a sharp interface.^[^ ^15^ ^]^ This combination ensures excellent charge transportation, paving the way for the fabrication of an efficient sensitive platform for SERS with a wider range of applications.^[^ ^9^, ^12^, ^17^ ^]^ In particular, composites based on the 2D material MoS_2_ have received a lot of attention from researchers.^[^ ^10^ ^]^ For example, MoS_2_/Au,^[^ ^18^ ^]^ MoS_2_/Ag,^[^ ^19^ ^]^ Cu/MoS_2,_^[^ ^20^ ^]^ D(C_7_CO)‐BTBT/MoS_2,_^[^ ^21^ ^]^ MoS_2_/Graphene,^[^ ^22^ ^]^ MoS_2_/graphene,^[^ ^23^ ^]^ W_18_O_49_/Monolayer MoS_2_,^[^ ^24^ ^]^ and MoS_2_/MoO_3_–x^[^ ^25^ ^]^ composites where such combinations not only take advantage of low cost, active edge sites, tunability, biocompatibility, high reproducibility, and more adsorption capability of MoS_2_ but also combine the properties of other materials, which form coupled enchantment of CM and EM working together.^[^ ^26^ ^]^ It further improves its limit of detection (LOD), stability, CT, sensitivity, uniformity, and selectivity characteristics as SERS substrate, which further expands its application in different areas. F_4_TCNQ is a small organic semiconductor with low lying lowest unoccupied molecular orbital (LUMO), strong electron affinity, effective electron acceptor, delocalized electrons, and good thermal stability.^[^ ^27^, ^28^ ^]^ Combining it with MoS_2_ will promote charge transfer, stability, more Raman scattering area, and increase density of state (DOS) near the Fermi level, and there will be an enhancement of the SERS activity of the substrate.^[^ ^29^, ^30^ ^]^

However, the SERS phenomenon concerned with hybrid organic/inorganic mainly relies on the CT mechanism, which is not too high. In order to further improve the SERS performance of such hybrid substrates, coupling CM with an intense electric field generated in AAO nanocavity.^[^ ^31^ ^]^ In this regard, nanoarchitectured AAO templates are effective in SERS activity due to the optical resonance inside the nanocavity (contributing to the EM mechanism). This template possesses excellent properties, such as high chemical and thermal stability and highly ordered structures with a large surface.^[^ ^32^, ^33^, ^34^, ^35^ ^]^ Consequently, an effective strategy to grow hybrid organic/inorganic heterostructures using an AAO template is to overcome issues related to metals and semiconductors and to increase the SERS substrate characteristics via CM and EM. Moreover, there will be an enhancement of photocatalytic degradation of molecules adsorbed on the surface of the substrate due to cavity photoconfined capability, which will optimize light‐matter interaction and cause enhanced CM. MoS_2_ can be used to make reusable SERS substrates but still suffers from reduced photocatalytic activity due to photocarrier recombination, which limits substrate recovery efficiency. Thus, the rational design of heterojunction‐based recyclable SERS substrates to increase recyclable efficiency should be investigated.^[^ ^36^ ^]^ Furthermore, such a combination of organic/inorganic materials opens up a novel approach to enhancing photocatalytic degradation activity. This is due to the effective surface hybridization with π‐conjugated systems, which broadens the light‐spectrum responsive range, extends the photogenerated charge lifetime, and enhances the stability of photocatalysts.^[^ ^37^ ^]^ Various combinations have been reported, including TiO_2_/porphyrin,^[^ ^38^ ^]^ CdS/NiPc,^[^ ^39^ ^]^ TiO_2_/Au/polythiophene,^[^ ^40^ ^]^ FePc/porous WO_3_,^[^ ^41^ ^]^ Cd/PDI,^[^ ^42^ ^]^ and WO_3_/polypyrrole.^[^ ^43^ ^]^ It is widely recognized that a catalyst with a larger specific surface area has a greater number of exposed active sites, leading to enhanced photocatalytic activity. Thus, in the context of practical applications, reusable SERS substrates offer fast, cost‐effective, and energy‐efficient removal of contaminants via organic/inorganic heterostructure, a widely adopted approach for photodegradation.

In this study, we combine the π‐conjugated organic semiconductor material F_4_TCNQ with MoS_2_ and utilize AAO templates to obtain a V‐shaped nanocavity heterostructured SERS substrate with a large specific surface area and highly ordered structure. The adopted AAO nanotemplates possess different depths of (400, 900, and 1500 nm) to explore how Al_2_O_3_ and AAO nanotemplates with different depths influence the SERS effect of the F_4_TCNQ/MoS_2_ heterostructure, using MB as a probe molecule. Highly sensitive SERS performance was observed at 1500 nm depth of AAO nanocavity compared to other AAOs for the first time, which was attributed to efficient charge transfer and more optical trapping. The F_4_TCNQ/MoS_2_/AAO (1500 nm) presents an ultra‐sensitive LOD of 9.0×10^−16^ M and has a high EF of 1.2 ×10^9^ for probe molecule MB. To the best of our knowledge, such sensitivity has not been documented previously via template‐assisted organic/inorganic semiconducting heterostructure. More importantly, the fabricated substrate exhibits reproducibility, stability, selective detection, and fast sensing. The efficient charge transfer of organic/inorganic semiconducting heterostructure enhances their photodegradation capability. Furthermore, the F_4_TCNQ/MoS_2_/AAO SERS platform can quickly and non‐destructively identify heavy metal ions (Hg^2+^) in water environments. These results highlight the significance of the combined effect of CT and EM phenomena. These features could be exploited for widespread applications, including Raman sensing in food safety, chemical analysis, and environmental monitoring. These key innovations are likely to generate significant interest among a wide audience from fields such as nanotechnology, materials science, and environmental science. Such innovative substrates can pave a new approach for constructing organic/inorganic heterostructures utilizing nanoarchitectured templates to achieve reusable and ultra‐sensitive SERS substrates for various practical applications.

## 2 Results and Discussion

### 2.1 Characterization of F_4_TCNQ/MoS_2_ and AAO Substrates

**Scheme 1:**
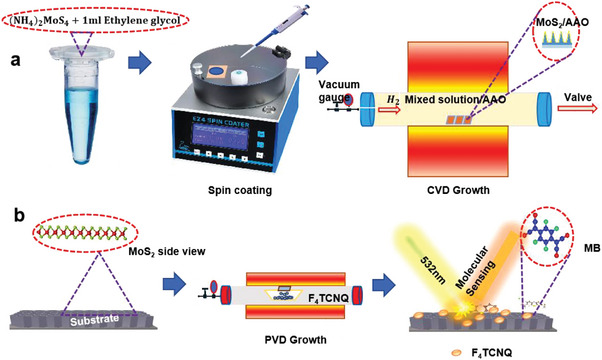
Overall experimental illustration of MoS_2_ and F_4_TCNQ. a) chemical vapor deposition of MoS_2_. b) physical vapor deposition of F_4_TCNQ on MoS_2_.

**Scheme** **1** illustrates a visual representation of the experimental procedure and the SERS performance of the F_4_TCNQ/MoS_2_/AAO heterostructure. First, a few‐layer MoS_2_ was successfully synthesized on three AAO substrates and one Al_2_O_3_ substrate using the ambient chemical vapor deposition (CVD) method. To grow the hybrid heterostructure of F_4_TCNQ and MoS_2_, a physical vapor deposition (PVD) process has been adopted within an evacuated furnace at a low pressure of 10^−6^ Torr. To ensure controlled deposition, the prepared MoS_2_ substrate was kept toward the organic powder in the boat covered by aluminum foil. A schematic visualization of the MoS_2_ and F_4_TCNQ growth on AAO with fixed top and bottom diameters of V‐shaped nanocavities is displayed in Figure S1 (Supporting Information). The curved surface of the V‐shaped channel enhances the intensity of EM and provides a large surface area for material deposition. To show the potential of our design, we fabricated the F_4_TCNQ/MoS_2_/AAO SERS substrate by successive deposition of MoS_2_ and F_4_TCNQ on AAO, respectively. It's worth noting that this approach aligns well with the existing standard large‐scale processing methods, leading to reliable and reproducible fabrication of SERS substrates. The morphology of the as‐prepared MoS_2_ on Al_2_O_3_ and three AAO nanotemplates with different depths was carried out through a scanning electron microscope (SEM). The top and side views of AAO are displayed in Figure S2 (Supporting Information), revealing the details of AAO's geometrical interpretation.

**Figure 1:**
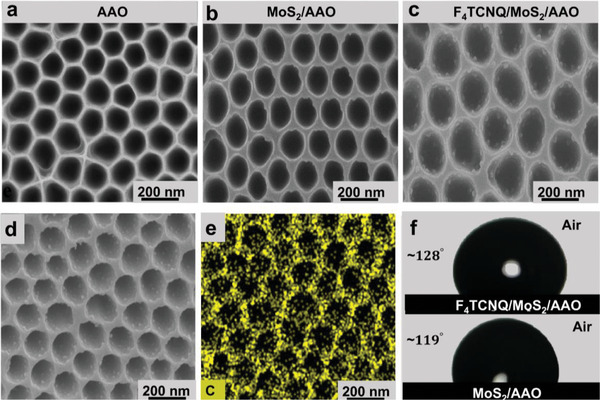
Structural and compositional analysis of F_4_TCNQ/MoS_2_ and AAO. (a‐c) surface morphology through SEM. a) bare AAO. b) MoS_2_/AAO, and c) F_4_TCNQ/MoS_2_/AAO (1500 nm). EDS analysis of d) F_4_TCNQ/MoS_2_/AAO. e) carbon. f) Water contact angle of F_4_TCNQ/MoS_2_/AAO and MoS_2_/AAO (1500 nm).

**Figure** **1**a‐c illustrates the top view of bare AAO, MoS_2_/AAO, and F_4_TCNQ/MoS_2_/AAO with 1500 nm depth, respectively. The grown MoS_2_ turned the hexagonal cell of the bare AAO into circular, organized nanocavities, confirming efficient growth. The deposited F_4_TCNQ nanoparticles on the MoS_2_ surface are disconnected and more concentrated into nanocavities with an average size of 39 nm. The distribution and concentration of F_4_TCNQ nanoparticles on the AAO templates differ from those on the Al_2_O_3_ substrate, as shown in Figure S3 (Supporting Information). The rest of the two AAO models with depths of 900 and 400 nm are displayed in the same manner as the AAO 1500 nm in Figure S4a–f (Supporting Information). The 400 nm AAO model's bottom is more visible compared to other AAO models. The top view diameters were found to be ≈450 nm on average for all three AAO templates. To measure the depth of the V channel and the bottom diameter of the nanocavity, the AAO substrate was broken at the midpoint and captured the morphology of the cross‐sectional view. As depicted in Figure S5 (Supporting Information), a consistent structure is observed through each template with varying depths (≈1500, 900, and 400 nm) and a nearly fixed bottom diameter ≈100 nm. To confirm the elemental composition and distribution within the F_4_TCNQ/MoS_2_ heterostructure, energy‐dispersive spectroscopy (EDS) mapping was generated. Figure 1d,e demonstrate the focused portion of F_4_TCNQ/MoS_2_/AAO and carbon components of F_4_TCNQ, respectively. Carbon shows a higher elemental distribution among others due to the higher molecular weight of carbon in F_4_TCNQ (C_12_F_4_N_4_), and the contribution confirms the disconnectivity among F_4_TCNQ nanoparticles depicted in Figure 1e. The EDS mappings of F, N, Mo, and S are highlighted with dark purple, indigo, dark orange, and green colors, respectively, as shown in Figure S6 (Supporting Information). Similarly, the EDS mapping of F_4_TCNQ/MoS_2_ on flat Al_2_O_3_ is displayed in Figure S7 (Supporting Information), which quantifies the uniformity of elemental composition and ensures the composite matches the desired stoichiometry. The wettability of F_4_TCNQ/MoS_2_/AAO is checked to evaluate the liquid droplet's interaction with the substrate surface, ultimately affecting the SERS enhancement.^[^ ^44^ ^]^ In this regard, the contact angle (CA) measurements of MoS_2_ and F_4_TCN/MoS_2_ are carried out for all four substrates. Among other AAO nanotemplates, the contact angle of water with MoS_2_ and F_4_TCN/MoS_2_ grown on AAO (1500 nm) is determined to be 117° and 128°, respectively, indicating the highest degree of hydrophobicity illustrated in Figure 1f. Due to the roughness of the AAO substrate and the presence of the F_4_TCNQ nitrile C ≡ N functional group, the liquid will form a high CA and will stay in a spherical droplet. The CA of water on the surface of MoS_2_ and F_4_TCNQ/MoS_2_ on AAO (900, 400 nm) are 103°, 118°, 89°, and 107°, respectively, shown in Figure S8 (Supporting Information). In the same manner, the CA demonstrated an increase in F_4_TCNQ/MoS_2_ (78°) compared to MoS_2_ (53°) grown on Al_2_O_3_, which is almost the same as previously reported for other plane substrates.^[^ ^45^ ^]^ Overall, an increasing trend was found for the CA with increasing depth of AAO (air trapping in V‐shaped nanocavities)^[^ ^46^ ^]^ and deposition of F_4_TCNQ (reducing surface energy).^[^ ^27^ ^]^ The more hydrophobic nature of AAO 1500 nm will lead to more molecules concentrating at a single point; hence, more SERS enhancement. The surface topography of the MoS_2_ and F_4_TCNQ/MoS_2_ organic/inorganic interfaces was investigated using atomic force microscopy (AFM), as detailed in the Supporting Information (Figure S9, Supporting Information).

**Figure 2:**
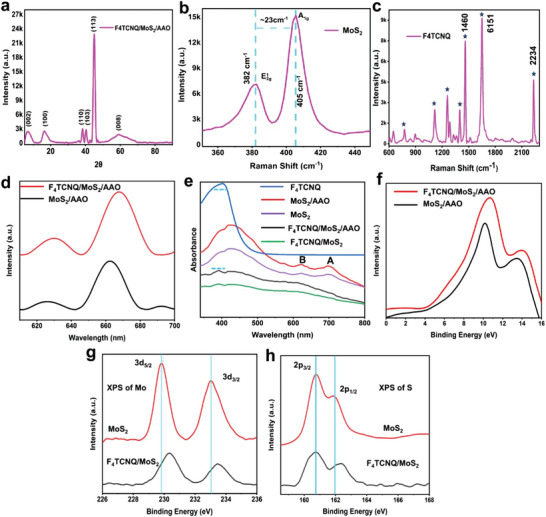
Optical and chemical compositional characterization of F_4_TCNQ/MoS_2_. a) X‐ray diffraction pattern of F_4_TCNQ/MoS_2_/AAO. b) Raman peaks of MoS_2_ are associated with out‐plane and in‐plane vibrations. c) Raman peaks of the F_4_TCNQ. d‐e) Photoluminescence and UV‐Vis absorption spectra of MoS_2_ and F_4_TCNQ/MoS_2_, respectively. f) UPS spectra of MoS_2_ and F_4_TCNQ/MoS_2_. XPS spectra of g) Mo and h) S before and after deposition of F_4_TCNQ, respectively.

The X‐ray diffraction (XRD) technique is employed to obtain a deep insight into the crystal structural characteristics of the combined material system F_4_TCNQ/MoS_2_/AAO. **Figure** **2**a displays the XRD pattern of F_4_TCNQ/MoS_2_/AAO with different diffraction peaks at 2θ = 7°, 15.7°, 38.4°, 40.4°, 45°, and 59°, corresponding to (002), (100), (110), (103), (113), and (008). Among these peaks, 100 correspond to F_4_TCNQ, characterized by a broad and moderately intense profile, which belongs to polymorphism 1, showing a poor alignment of the crystal shown in Figure S10 (Supporting Information).^[^ ^47^, ^48^ ^]^ This peak, 2θ = 15.7° (d = 5.639 Å), corresponds to F_4_TCNQ, characterized by a slightly broad and moderately intense profile, which belongs to polymorphism 1, showing a poor alignment of the crystal shown in Figure S10 (Supporting Information).^[^ ^48^ ^]^ This phase highlights the orientation of the herringbone configuration of molecules along (100) and the interaction between molecules in neighboring layers. The F_4_TCNQ molecules moved and diffused less, which caused nanoparticles to form on the substrate surface. This made the (100) peak in XRD less intense and wider owing to the fact that F_4_TCNQ on the substrate had changed into a different phase. Further, inconsistencies exist between the lattice constant of the single crystal and the d‐spacing of F_4_TCNQ, suggesting that the phase of the organic material is distinct from the single crystal phase. The planes (002), (103), and (008) corresponding to MoS_2_ are well‐matched with the MoS_2_ XRD profile.^[^ ^49^ ^]^ The broad peak of MoS_2_ is due to the nonuniform strain arising from the F_4_TCNQ. The remaining two crystallographic planes (110) and (113) are attributed to AAO, with sharp peak patterns showing rich periodicity.^[^ ^50^ ^]^

Raman and photoluminescence (PL) spectra were utilized for the analysis of sample quality and optical properties. Figure 2b displays the Raman spectra of MoS_2_/AAO, with two characteristic peaks of E^1^_2g_ (382 cm^−1^) and A_1g_ (405 cm^−1^) corresponding to in‐plane and out‐plane vibrations of the Mo and S atoms.^[^ ^51^ ^]^ Furthermore, it has been demonstrated that the frequency difference between these two modes can serve as a reliable means to differentiate between few‐layer MoS_2_ and its bulk form while estimating the number of monolayers present. The present work observed a frequency difference of 23 cm^−1^ for the MoS_2_ trilayer. The sharp intensity of Raman peaks was observed for the 1500 nm AAO model, attributed to more optical resonance and higher photon density, leading to strong Raman scattering in the deep V‐shaped nanocavities. To consider the substrate's impact, Figure S11 (Supporting Information) compares the grown MoS_2_ Raman peaks on four different substrates. It is evident that AAO (1500 nm) exhibits a higher photon density, leading to stronger light‐matter interactions. The Raman spectra of F_4_TCNQ have two prominent peaks at 1640 cm^−1^ and 1450 cm^−1^, corresponding to the C‐N stretching, C ═ C ring stretching, and C‐C in‐plane stretching modes, respectively,^[^ ^45^ ^]^ as depicted in Figure 2c. The C‐N (2234 cm^−1^) stretching mode will help to differentiate between a probe molecule and F_4_TCNQ because most of the probe molecules possess peaks ≈1620 cm^−1^.^[^ ^52^ ^]^ After successful deposition of F_4_TCNQ on MoS_2_, a significant change was observed in the Raman spectra of MoS_2_, as shown in Figure S12 (Supporting Information). This shift may be attributed to the strain due to the F_4_TCNQ nanoparticle and charge transfer, which can change the electronic structure. The PL spectra of MoS_2_ and F_4_TCNQ/MoS_2_ are shown in Figure 2d. The few layers of MoS_2_ show vigorous PL intensity compared to the F_4_TCNQ/MoS_2_ heterostructure, indicating some non‐radiative recombination. The MoS_2_ PL spectra consist of two major peaks at 670 nm and 627 nm, attributed to A and B excitons associated with a direct transition at the high‐symmetry K‐point.^[^ ^53^, ^54^ ^]^ The difference between these excitons is caused by the valence band (VB) splitting resulting from strong spin‐orbit coupling. The emission is likely to occur more from exciton A compared to B. After combining MoS_2_ with F_4_TCNQ, a blue shift is observed in the PL spectra, with the appearance of an additional peak at lower energy corresponding to F_4_TCNQ. This decrease is due to the appearance of a new energy level in the band structure.^[^ ^55^ ^]^

To gain further insight into the optical properties of the organic/inorganic heterostructure, it is important to consider that 2D materials offer exciting possibilities; however, a major drawback is their inherently weak light absorption due to their atomically thin nature.^[^ ^56^ ^]^ A generally adopted approach is placing the 2D material within a cavity structure to overcome this limitation and achieve strong light‐matter interaction, which leads to dramatically enhanced absorption. Our results demonstrate in Figure 2e, that the introduction of the AAO i.e., MoS_2_/AAO (red color) indeed leads to enhanced UV‐Vis spectra than MoS_2_ without AAO (purple color).^[^ ^56^ ^]^ The UV‐Vis absorption spectra of MoS_2_ reveal the presence of two significant peaks, A and B, at 695 and 623 nm, respectively.^[^ ^57^ ^]^ These absorption peaks result from the VB splitting due to strong orbit coupling at the K‐point in the Brillouin zone. After depositing F_4_TCNQ, the intensities of these peaks show a slight decrease, with an additional peak appearing at 397 nm corresponding to F_4_TCNQ shown in Figure 2e.^[^ ^58^ ^]^ Furthermore, compared to F_4_TCNQ/MoS_2_ (green color), the F_4_TCNQ/MoS_2_/AAO (black color) exhibits improved absorption spectra, demonstrating how AAO nanocavities enhanced the absorption spectra. This observation can be attributed to the increased light‐matter interaction facilitated by the high surface area and cavity's resonant properties that trap light.^[^ ^56^ ^]^ A comparison between Al_2_O_3_ and AAO has been carried out to further verify the influence of the cavity structure on the enhanced absorption spectra. The AAO exhibits enhanced absorption compared to that of Al_2_O_3_ resulting in a more resonance effect leading to more light interaction, as shown in Figure S12b (Supporting Information).^[^ ^59^ ^]^ Moreover, the F_4_TCNQ solution exhibits a major absorption peak at 402 nm (blue color) presented in Figure 2e.^[^ ^58^, ^60^ ^]^ From the above results, it can be concluded that cavity structure enhances the absorption of light, i.e., more light‐matter interaction (resonance effect), which is important for high SERS performance due to more electromagnetic enhancement and charge transfer.

The charge transfer at the F_4_TNCQ/MoS_2_ interface was confirmed by utilizing ultraviolet photoelectron spectroscopy (UPS). The UPS spectra of organic/inorganic composite (red color) shifted toward higher energy and high intensity compared with pristine MoS_2_ (black color) shown in Figure 2f, confirming the formation of the F_4_TNCQ/MoS_2_ interface. This shift suggests more electrons were transferred from MoS_2_ to F_4_TCNQ (the electron acceptor) and an increase in the DOS near the Fermi level, which is critical for efficient SERS performance.^[^ ^61^ ^]^ The strong electron‐withdrawing property of F_4_TCNQ induces electron transfer from MoS_2_ to F_4_TCNQ, resulting in p‐type doping of MoS_2_. This phenomenon is corroborated by an upward band‐bending of MoS_2_‐related electronic states along with an increasing work function compared to pristine due to the transfer of the valence. The UPS spectrum of the F_4_TCNQ/MoS_2_ composite reveals a pronounced enhancement in spectral intensity compared to pristine MoS_2_, suggesting a substantial degree of hybridization between the organic and inorganic components. This increased density of states near the Fermi level, as evidenced by the UPS data, is a critical factor in augmenting the SERS performance of the composite material.^[^ ^61^ ^]^

X‐ray photoelectron spectroscopy (XPS) was conducted to analyze the surface chemical states and chemical shifts of pristine MoS_2_ and the F_4_TCNQ/MoS_2_ heterostructure. To address this, we focused on the Mo 3d core level spectrum. The Mo 3d spectrum typically exhibits a doublet consisting of Mo 3d_5/2_ and Mo 3d_3/2_ peaks, separated by a spin‐orbit splitting of ≈3.2 eV. The binding energy of the Mo 3d peaks can provide information about the oxidation state of Mo. A shift to higher binding energy generally indicates a higher oxidation state. Figure 2g displays the core‐level XPS spectra of Mo 3d in pristine MoS_2_ at binding energies of 229.95 eV and 233.45 eV, corresponding to (Mo 3d_5/2_) and (Mo 3d_3/2_), respectively, due to spin‐orbit splitting by (Δ*E*) of 3.3 eV.^[^ ^62^ ^]^ Such peaks correspond to the existence of the MoS_2_ 2H phase that confirms the Mo ^4+^ state.^[^ ^63^ ^]^ This means that after the deposition of F_4_TCNQ, the Mo valence state partially oxidized, which shows more affinity toward the probe molecule, which is important for SERS. The S 2p doublets, 2p_1/2_ and 2p_3/2_ are observed at 160.8 eV and 162.1 eV, respectively. The peak positions and splitting energy of 1.3 eV correspond to the −2 oxidation state of S in the Mo–S bonds of MoS_2_.^[^ ^63^ ^]^ The deposition of F_4_TCNQ induces a chemical shift toward higher binding energy in the case of Mo, where two core‐level peaks belong to a shift of ≈ 0.5 eV. The same tendency of chemical shift is observed in the case of S, where the binding energy peaks of 2p_3/2_ and 2p_1/2_ are located at 160.8 eV and 162.1 eV, respectively, as shown in Figure 2h.^[^ ^64^ ^]^ The electron transfer from MoS_2_ to F_4_TCNQ after the formation of F_4_TCNQ/MoS_2_ interfaces shifts the Fermi level of MoS_2_ downward toward its valence band maximum (VBM), leading to upward band‐bending in MoS_2_. This upward band‐bending at the interface is confirmed by our XPS results. Due to the strong electron‐withdrawing nature of F_4_TCNQ,^[^ ^28^ ^]^ electrons transfer from MoS_2_ to F_4_TCNQ at the interface, which alters the chemical environment of Mo and S atoms. The remaining electrons experience a stronger electrostatic pull toward the positively charged Mo and S nuclei. This strong attraction is reflected in the higher binding energy shift of both the Mo and S peaks. The Fermi level of F_4_TCNQ/MoS_2_ shifted toward the VB of the resultant band gap in comparison to pristine MoS_2_, as shown by the decrease in binding energies of C 1s, F 1s, and N 1s, which correspond to 283.7 eV, 686.1 eV, and 397 eV, respectively, shown in Figure S13 (Supporting Information).^[^ ^65^ ^]^

### 2.2 Evaluation of SERS Activity and its Mechanism

**Figure 3:**
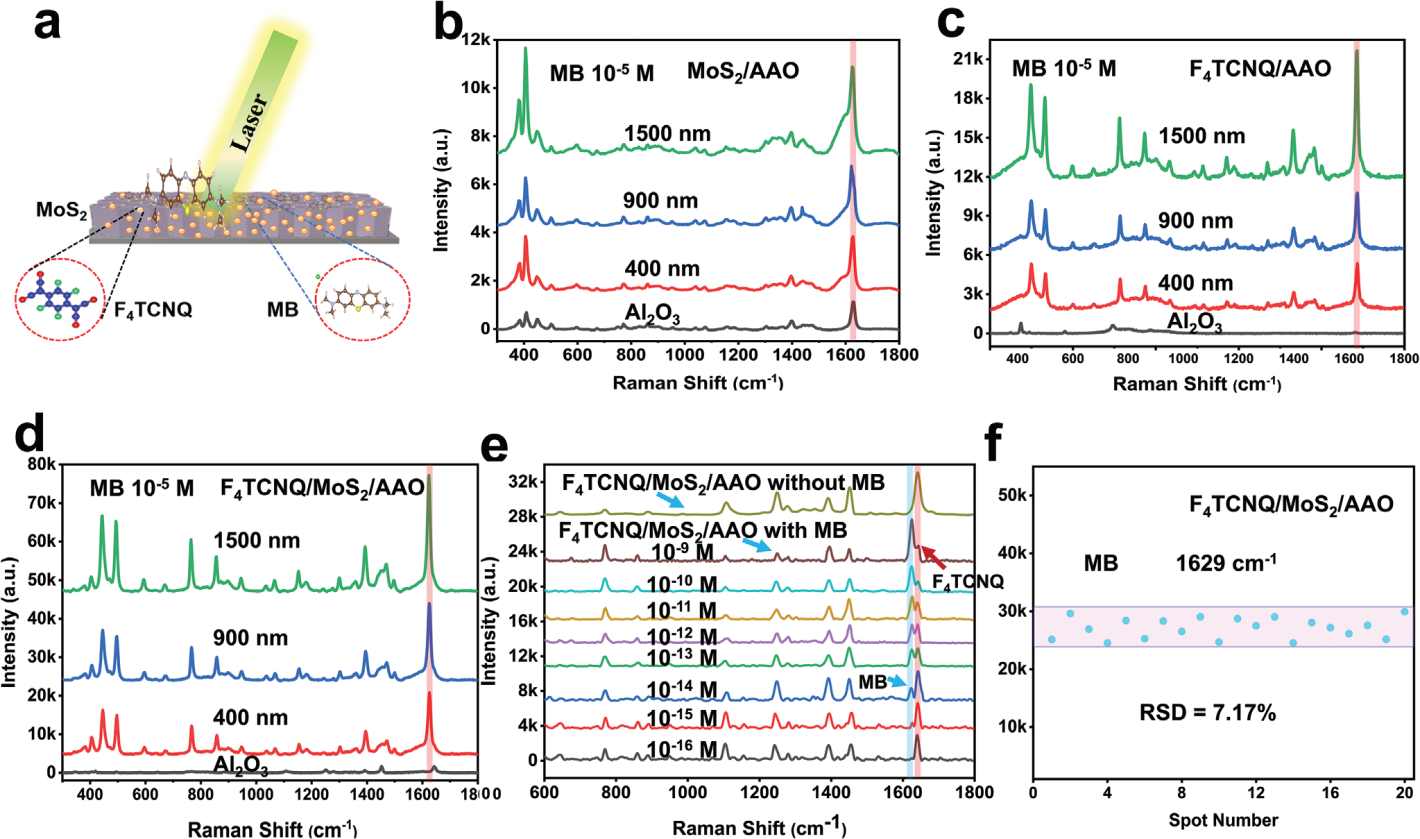
SERS effect of different substrates. a) Schematic illustration of MB on the grown substrate under a 532 nm laser. Raman spectra of 10^−5^ M MB on b) MoS_2_/F_4_TCNQ. c) F_4_TCNQ/AAO. d) F_4_TCNQ/MoS_2_/AAO. e) The Raman spectra of F_4_TCNQ/MoS₂/AAO and MB at different concentrations on the F_4_TCNQ/MoS₂/AAO substrate. f) RSD of the MB peak at 629 cm^−1^.

It is widely acknowledged that SERS enhancement arises from EM, CM, or a combination of both. Chemical enhancement is important in semiconducting materials due to the vibronic coupling of CT transitions between the substrate‐analyte system's aligned energy levels, which resonate with the incident laser. A schematic Raman scattering of MB molecules on the F_4_TCNQ/MoS_2_ heterostructure stimulated by a 532 nm laser is depicted in **Figure** **3**a. The study aimed to explore how Al_2_O_3_ and AAO nanotemplates with different depths influence the SERS effect of the F_4_TCNQ/MoS_2_ heterostructure, using MB as a probe molecule. The Raman spectra of probe molecule MB at 10^−5^ M concentration were collected from four samples: bare Al_2_O_3_ and AAO (400, 900, and 1500 nm) nanotemplates, as shown in Figure S14 (Supporting Information). Notably, the Raman intensity of the prominent MB peak was hardly detectable on the bare Al_2_O_3_ substrate. However, this peak's intensity increased significantly as the AAO's depth increased. The most intense Raman spectra of MB can be achieved through AAO with a depth of 1500 nm due to its more light‐capturing capability.^[^ ^66^ ^]^ Figure 3b illustrates the Raman peaks of MB at a concentration of 10^−5^ M of MoS_2_ grown on different substrates, and a comparison of the 1629 cm^−1^ peak of MB among different substrates is displayed in Figure S15 (Supporting Information). This comparison facilitates the evaluation of how the substrate material influences the Raman signal of MB. The effectiveness of each substrate in enhancing the Raman signal can be determined by comparing the intensity or any alterations in the peak position among different substrates. Two distinct peaks are observed at ≈382 cm^−1^ and 405 cm^−1^ for MoS_2_, and a prominent peak at 1629 cm^−1^ for MB. For both MoS_2_ and MB, the enhanced spectra arise from the deepest AAO among the other AAO substrates. Figure 3c,d illustrate the evolution of Raman spectra for MB molecules adsorbed on the grown F_4_TCNQ and F_4_TCNQ/MoS_2_, respectively. The MB and F_4_TCNQ peaks slightly shift toward each other, merging at a higher concentration of the probe molecule. The same pattern was observed for the as‐grown F_4_TCNQ/MoS_2_, with higher intensity than F_4_TCNQ. When excited by the Raman laser, the amplified Raman peaks can be attributed to the excellent CT mechanism between F_4_TCNQ/MoS_2_ and MB molecules.^[^ ^45^ ^]^

**Figure 4:**
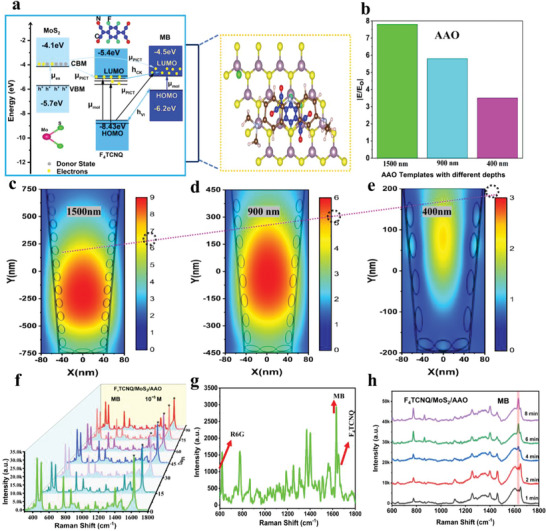
Mechanism of SERS activity based on charge transfer and electromagnetic enhancement of F_4_TCNQ/MoS_2_/AAO. a) CT mechanism of F_4_TCNQ/MoS_2_/AAO and MB molecules. b) Bar graph of EM values with respect to different nanocavity depths of AAO. EM enhancement inside the V‐shaped nanocavities of AAO nanotemplates with different depths. c) 1500 nm, d) 900 nm, e) 400 nm. f) Stability. g) Mixed solution of MB and R6G. h) SERS response with respect to time.

The MB peaks on F_4_TCNQ/MoS_2_ experience significant alterations in both intensity and location, suggesting strong adsorption of MB onto the as‐prepared heterostructure, which can promote the possibility of an efficient electron transition between them. In the F_4_TCNQ/MoS_2_ system, the highest occupied molecular orbital (HOMO) and LUMO of MB are located at −6.2 and −4.5 eV, respectively.^[^ ^27^ ^]^ The F_4_TCNQ/MoS_2_ system can be excited by a 532 nm laser to induce Raman scattering by exciting the molecular transition (µm_ol_) and exciton transition (µ_ex_). The conduction band minima of MoS_2_ are higher than those of F_4_TCNQ's LUMO, with more electron donor states that facilitate photo‐induced charge transfer (PICT). The initial electron transfer from MoS_2_ to F_4_TCNQ helps in the separation of the photogenerated electron‐hole pair in MoS_2_, potentially increasing the lifespan of the excited state. This can be advantageous for SERS since it provides more time for the Raman scattering process to begin. This extraction creates a space charge region at the interface, where transferred charge accumulates and causes Fermi level shifting. Electrons become immobilized within separated organic particles, while at the same time, holes localized at the donor states in MoS_2_ undergo strong localization. Usually, semiconductor‐based SERS substrates consist of randomly distributed, disconnected nanoparticles on the substrate surface. Constructing the combined interface of MoS_2_ and F_4_TCNQ leads to an increase in the DOS near the Fermi level, narrowing the band gap, and hence enabling improved CT efficiency, as confirmed by theoretical calculations. This increased DOS has a linear relationship with the CT process, in accordance with the Fermi golden rule.^[^ ^67^ ^]^ **Figure** **4**a shows the PICT routes between the F_4_TCNQ/MoS_2_ substrate and the MB probe molecule. Further, the increased photogenerated carriers in F_4_TCNQ LUMO result in a strong electric field to polarize the MB. The MB molecules exhibit the highest Raman intensity for F_4_TCNQ/MoS_2_ because the 532 nm laser has the closest energy to the energy level difference between the LUMO and HOMO of F_4_TCNQ. An additional energy level appears in the band gap of the combined system due to electron transfer revealed by density functional calculation, confirming the decrease in PL intensity.^[^ ^29^ ^]^ Whenever the excitation source has energy above the band gap of the material, the initially excited hot valence electrons with higher energy transfer into the conduction band, where the thermalization process occurs by e‐e scattering or electron‐phonon scattering.^[^ ^68^, ^69^ ^]^ These hot electrons then transfer from MoS_2_ to F_4_TCNQ and MB via PICT due to relatively same energy level positions leading to more charge transfer upon excitation. Thermodynamically permissible charge transfer can occur either from a semiconductor to a molecule or from a molecule to a semiconductor.^[^ ^69^ ^]^ The thermodynamically possible charge transfer mechanism resulting from the activation of a 532 nm laser involves the PICT resonance from the conduction band of MoS_2_ to the LUMO level of F_4_TCNQ and MB due to their relative same position. Thus, a charge transfer takes place from MoS_2_ to MB and F_4_TCNQ in the context of PICT facilitated by the excitation with a 532 nm laser as shown in Figure 4a. The PICT transition from F_4_TCNQ HOMO to MB LUMO enhances the SERS effect through charge transfer resonance. This can borrow intensity from the molecular and exciton transitions through the Herzberg‐Teller coupling constants (h_VI_ and h_CK_), making the probe molecules more polarized and increasing the Raman scattering cross‐section.^[^ ^70^ ^]^ The ground‐state electrons of MB molecules can be stimulated by laser light (2.33 eV) to reach excited‐state levels. Recombining excited‐state carriers to the ground or lower levels can increase probe molecular polarization and the Raman scattering cross‐section of MB. Additionally, the PICT amplifies the Raman signal of MB molecules on the F_4_TCNQ/MoS_2_ substrate by two mechanisms. F_4_TCNQ/MoS_2_ nanocomposite valence band charges can be stimulated to the conduction band and transported to molecular levels above the LUMO of MB, enhancing the MB molecular Raman signal. The studies indicate that the µm_ol,_ µ_ex,_ and PICT procedures enhance the SERS activity of the F_4_TCNQ/MoS_2_ nanocomposite substrate. Besides CT contributions from incident light absorption, EM enhancement was also investigated to check the impact of nanocavity on SERS optimization. Furthermore, when ordered structures are introduced on a sub‐wavelength scale, additional photon‐matter interactions come into play, including reflectance, resonance, and interference.^[^ ^71^ ^]^ The cavity structure of AAO leads to an increase in the scattering intensity of target molecules. An increasing trend is found as the nanocavity depth increases, highlighted by black dashed circles with |E/E_o_| 2.8, 5.7, and 6.9 for 400, 900, and 1500 nm, respectively, comparison is shown in Figure 4b. The strongest electric field was observed for the 1500 nm AAO substrate. Figure 4c‐e provides essential insights into the relationship between the electric field intensity ratio (E/E_o_) and the distribution of electric fields. The normalized electric field amplitude, represented by |E/E_o_|, depicts the intensity of the electric field. Here, E_o_ referred to the incident electric field intensity, while E represented the enhanced local electric field intensity. It is found that simple AAO and MoS_2_/AAO have less electric field intensity compared to F_4_TCNQ/MoS_2_/AAO, as displayed in Figure S16 (Supporting Information). To get a deep insight into the nanocavity impact on EM, Purcell factors (P_F_) play a crucial role in understanding the physical process of V‐shaped nanocavities.^[^ ^71^ ^]^ Such cavities can be **c**haracterized by a high‐quality factor (Q) and a mode confined within an ultra‐small volume (V), which significantly enhances the density of photon states. Mathematically, the Purcell factor is defined as:

(1) $$P_{F}=\left(3/4\pi^{2}\right)\left(Q/V_{\mathrm{mode}}\right){\left(\lambda /2n\right)}^{3}$$

where λ represents the wavelength and n signifies the refractive index, which is an important measure of the increase in photon state density within the resonant nanocavity. Additionally, the Raman gain (G_R_), defined as the ratio of the power of the Raman signal radiated in the presence of the resonant structure to the power radiated in its absence, is an essential term for universal description in complex systems, as proposed recently.^[^ ^72^ ^]^ This highlights the significance of designing cavities with high PF for improving the performance of SERS. In addition, a recent study examined the enhanced Raman signal by increasing the depth of the V‐grooves nanoarray.^[^ ^71^ ^]^ This allowed the researchers to design cavities that can sustain numerous resonant modes with varying depths. Moreover, adjusting the V‐shaped depth enables AAO nanocavities to support multiple coupling modes, enhancing Raman signals and leading to crucial practical applications of SERS. This indicates that increasing the depth of nanocavities results in a substantial increase in electromagnetic enhancement due to more light‐matter interaction within the AAO nanocavities. Further, optimizing the nanocavity depth and incorporating suitable organic/inorganic heterostructures within the AAO nanotemplate increase EM enhancement, thereby improving the SERS signal. Furthermore, the contribution to the gradual enhancement of Raman signals on each AAO substrate is due to the intense electric field inside the AAO nanocavities with increasing depth. These results demonstrate the effective control of depth variations of V‐shaped nanocavities on the SERS effect.

A series of solutions containing MB with varying concentrations, spanning from 4.0 × 10^−4^ to 9.0 × 10^−16^ M, were precisely prepared through a dilution process to enable the detection of trace amounts of these molecules. Remarkably, the Raman peaks of MB on F_4_TCNQ/MoS_2_/AAO (1500 nm) remained detectable even at ultralow concentrations of 9.0 × 10^−16^ M (as depicted in Figure 3e). Compared to F_4_TCNQ/MoS_2_/AAO (1500 nm), 6L‐NbSe_2_ can also reach up to 5×10^−16^ M for Rhodamine 6G.^[^ ^73^ ^]^ However, 6L‐NbSe_2_ exhibits a metallic nature. Our strategy is to emphasize how we will bring semiconducting materials comparable to metal or even better regarding SRES sensitivity. Achieving a SERS sensitivity of 9.0×10^−16^ M with a semiconducting material is noteworthy and significant; obtaining such sensitivity with a semiconducting substrate underscores the effectiveness of our novel substrate. To make a clear distinction between the MB and F_4_TCNQ peaks at ≈1600 cm^−1^, the Raman spectra of F_4_TCNQ/MoS_2_/AAO with and without MB adsorption are plotted in Figure 3e. In the case of the Raman spectra of F_4_TCNQ/MoS_2_/AAO (depicted in gray), there is no characteristic MB peak at 1629 cm^−1^. Upon MB adsorption onto F_4_TCNQ/MoS_2_/AAO, the prominent MB peak at 1629 cm^−1^ becomes visible, located near the F_4_TCNQ peak at 1651 cm^−1^, as highlighted by the vertical cyan and red lines, respectively, in Figure 3e. This figure provides a clearer comparison of the Raman spectra of MB on the SERS substrates and F_4_TCNQ peak, which helps to accurately distinguish between the Raman peaks of substrate and probe molecule. In Figure 3e, the Raman spectra for various concentrations of MB (from 10^−9^ M to 10^−16^ M) on the F_4_TCNQ/MoS_2_/AAO substrate are presented. The prominent peak at 1629 cm^−1^, consistently observed across different concentrations highlighted by a vertical line (light cyan color), is attributed to MB. This is distinct from the characteristic peaks of F_4_TCNQ. The consistency and intensity of the MB peak at 1629 cm^−1^, even at the lowest concentration (10^−16^ M), confirm the detection of MB molecules and validate the reported LOD. To further clarify this lowest concentration, Raman mapping is a highly effective tool for identifying specific molecules over a large scanned area, as discussed and displayed in Supporting information (Figure S17, Supporting Information). At higher concentrations, most of the peaks merge to due their high charge transfer. In Figure 3e we can see the MB peak (1629 cm^−1^) with the increase in concentration it goes up compared to that of F_4_TCNQ but when we decrease the concentration the F_4_TCNQ peaks become more dominant at 1651 cm^−1^. After dropping MB on the surface of the F_4_TCNQ/MoS_2_ heterostructure, the assigned Raman vibrational peaks of MB revealed slight broadness and shifts in the positions (see Table S1, Supporting Information). The results suggest that the differences between the HOMO and the LUMO of F_4_TCNQ decreased, leading to a more effective charge transfer process confirmed by UPS results and more light‐matter interaction within the AAO nanocavity. The charge and energy delivered to the stretching modes of MB molecules are different inside the V‐shaped nanocavities and on the surface. To the best of our knowledge, this achievement is a noteworthy milestone since it is the first time that such an ultralow LOD for probe molecules has been achieved on the F_4_TCNQ/MoS_2_ heterostructure assisted by a nanoarchitectured template in comparison to other substrates with or without nanotemplates (as detailed in Table S2 in the Supporting Information). As a result, F_4_TCNQ/MoS_2_/AAO at 1500 nm stands out as an effective SERS platform with ultra‐high sensitivity for molecular sensing. The enhancement factor calculation (supporting information) for MB on F_4_TCNQ/MoS_2_/AAO 1500 nm is estimated to be 1.2 × 10^9^, with bulk molecules serving as the reference (as shown in Figure S18, Supporting Information).

In the context of practical applications of SERS, ensuring the reproducibility of measured data is of critical consideration. One key factor in achieving high reproducibility is the homogeneity of the Raman signal obtained from different substrates. The commonly employed metric for evaluating reproducibility is the relative standard deviation (RSD) of the Raman signal intensity less than 20% in SERS measurements over a micrometer‐sized area indicating high reproducibility.^[^ ^74^ ^]^ Here, five batches of F_4_TCNQ/MoS_2_/AAO were chosen to evaluate reproducibility under the same experimental conditions, with each sample contributing five spectra. In total, twenty spectra from distinct batches of the proposed substrates exhibit consistency and display the same characteristics as the peaks displayed in Figure S20a (Supporting Information). Moreover, twenty different measurements were carried out to find the reproducibility of the individual peak at 1629 cm^−1^ of MB, for which RSD was calculated by the following equation:

(2) $$\mathrm{RSD}\;=\frac{\sqrt{\frac{\sum_{i=1}^{n}{\left(I_{i}-\bar{I}\right)}^{2}}{n-1}}}{\bar{I}}$$

where I_i_ is the SERS peak intensity collected from each point at 1629 cm‐1, $\bar{I}$ represents the average of all SERS peak intensities, and n is the number of measured spectra, which is 20 in this case. The RSD value was found to be 7.17%, revealing the good reproducibility displayed in Figure 3f. The uniformity of the SERS substrate was initially assessed through point‐by‐point SERS mapping. Figure S20b (Supporting Information) illustrates the SERS mapping image of the F_4_TCNQ/MoS_2_/AAO based on the intensity of the MB characteristic peak at 1629 cm^−1^ over a 40 µm × 40 µm area. This signifies a high uniformity of the innovative SERS substrate confirmed by Raman mapping. In addition to high sensitivity and reproducibility, stability is a critical parameter for assessing the performance of SERS‐active substrates. Metallic/semimetallic transition metal dichalcogenides excel in probe sensitivity for dye molecules, but they often suffer from poor stability over extended periods, limiting their practical utility as SERS substrates (Table S2, Supporting Information). To evaluate the stability of the F_4_TCNQ/MoS_2_/AAO substrates, MB detection was performed at a concentration of 10^−5^ M every 15 days. Figure 4f presents the Raman spectra of MB obtained from the F_4_TCNQ/MoS_2_/AAO substrate at different detection times. The calculation of the peak intensity at 1629 cm^−1^ revealed a slight decrease in Raman signal intensity over 90 days. This result indicates that our F_4_TCNQ/MoS_2_/AAO (1500 nm) substrate exhibits outstanding reproducibility and stability. This stability is notably superior to that of the 2D transition metal dichalcogenides like 1T’‐MoTe, W_18_O_49_/MoS_2_, and NbS_2_ (see Table S2, Supporting Information), underscoring the practical applicability and durability of the F_4_TCNQ/MoS_2_/AAO (1500 nm) substrate for long‐term use. To check universality, a solution containing both R6G and MB of the same concentration (5 × 10^−6^ M) was mixed to investigate the selective detection capabilities of the sample, as shown in Figure 4g. Simultaneously, the F_4_TCNQ/MoS_2_/AAO substrate exhibits excellent versatility in detecting different molecules. Ultra‐rapid detection is another key aspect of SERS applicability, with notable benefits in real‐time observation and molecular analysis. In general, detection within 5 to 15 min is widely considered fast sensing.^[^ ^75^ ^]^ The rapid response with respect to the time of the grown substrate was assessed for the MB probe molecule. The SERS spectra of MB at a concentration of 10^−6^ M were collected from the F_4_TCNQ/MoS_2_/AAO for different time intervals (1–8) min, as shown in Figure 4h. Both the CM and EM contribute to the overall SERS signal in this case. Consequently, one can observe the MB peak in seconds. As time increases, more and more atoms accumulate on the hydrophobic surface, resulting in more charge transfer and more light‐matter (molecule) interaction inside the V‐shaped nanocavities of AAO. Our substrate validates the fast sensing due to its high affinity for target molecule adsorption and its cavity‐like structure providing increased surface area. This design minimizes the time required for the analyte to interact with the SERS active regions, allowing detection to occur within one minute. In the context of ultra‐rapid response, the as‐prepared substrate has shown excellent performance, which plays a significant role in practical applications.

### 2.3 Photocatalytic Degradation

**Figure 5:**
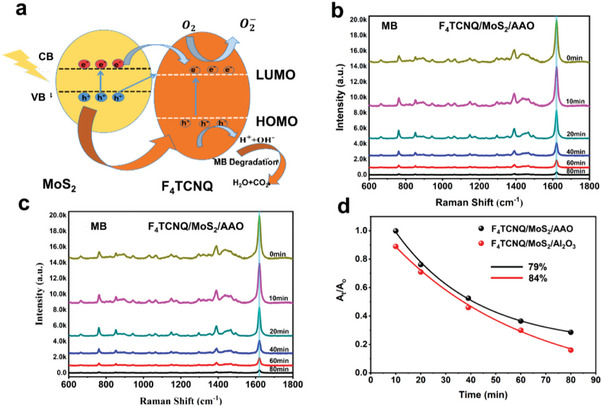
a) Schematic of photodegradation of MB. Photodegradation activity of F_4_TCNQ/MoS_2_ on b) Al_2_O_3_, and c) AAO. d) degradation rate of MB through F_4_TCNQ/MoS_2_/AAO and F_4_TCNQ/MoS_2_/Al_2_O_3._

Photocatalytic degradation is the most common way to recover SERS substrates because it eliminates contaminants quickly, economically, and efficiently. These methods are eco‐friendly since they avoid solvents and chemical waste. Photocatalytic degradation mainly relies on semiconductor‐like materials with CT capabilities and an energy gap between the VB and conduction band (CB). The CT between semiconductor nanomaterials and molecules depends on VB or CB vibrational coupling to the excited and ground states of the molecule. Adsorbed molecules excite from HOMO to LUMO energy levels. Single‐component photocatalysts, whether inorganic or organic nano‐semiconductors suffer from rapid charge recombination due to short transfer paths. Heterojunction photocatalysts may have significant potential due to their enhanced charge separation efficiency, increased light absorption, built‐in electric field, and robust redox potential.^[^ ^76^ ^]^ The current study proposes an innovative approach for producing heterojunction‐based recyclable SERS substrates with high photocatalytic efficiency and SERS performance. A schematic diagram for the photocatalytic degradation of MB through the F_4_TCNQ/MoS_2_ heterostructure is shown in **Figure** **5**a. Under UV‐vis illumination of the heterostructure interface, the photogenerated electrons transfer from the VB to the CB of MoS_2_ and HUMO to the LUMO of F_4_TCNQ, creating vacancies (holes). MoS_2_ and F_4_TCNQ possess band gaps of 1.78 eV and 3.03 eV, respectively, due to the potential difference in Photogenerated electrons prefer to move from MoS_2_ CB to F_4_TCNQ LUMO, as shown in the schematic diagram. The holes convert moisture in the air into hydroxyl radicals (*OH* ^−^) and electrons form superoxide radicals $\left(O_{2}^{-*}\right)$ by interacting with surface oxygen molecules. Thus, such a heterojunction inhibited photoexcited carrier recombination to increase electron migration and radical production. These radicals attack to decompose adsorbed MB molecules on the F_4_TCNQ/MoS_2_ nanocomposite. The following equations represent photocatalytic degradation series interactions;^[^ ^77^ ^]^ below are the reaction steps and formulae for F_4_TCNQ/MoS_2_ photocatalytic degradation.

(3) $$F_{4}\mathrm{TCNQ}/MoS_{2}+hv\;\to \;e_{CB}^{-}+h_{VB}^{+}$$

(4) $$e^{-}+O_{2}\to \;O_{2}^{-*}$$

(5) $$h^{+}+H_{2}O\to \;H^{+}+OH^{-}$$

(6) $$h^{+}+OH^{-}\to \;OH^{*}$$

(7) $$O_{2}^{-*}+2H^{+}\to \;2H_{2}O_{2}$$

(8) $$H_{2}O_{2}+O_{2}^{-*}\;\to OH^{-}+OH^{*}+O_{2}$$

(9) $$H_{2}O_{2}+e^{-}\;\to \;OH^{-}+OH^{*}$$

(10) $$OH^{-}+OH^{*}+dye\;\left(MB^{*}\right)\to +CO_{2}+H_{2}O$$

Demethylation, breaking the core aromatic ring of the MB structural bond, and breaking the side aromatic rings are all stages of MB degradation. Certain generated molecules, such as carboxylate, aniline, phenol, and R‐NH3, can be converted into other species like CO_2_, H_2_O, NO_3_, SO_42_−, and NH_4_.^[^ ^77^ ^]^ In Figure 5b, the F_4_TCNQ/MoS_2_/Al_2_O_3_ heterostructure demonstrates the photocatalytic degradation rate of the MB over 60 min with a 12‐minute interval between each cycle. After five cycles, the photodegradation of MB dropped significantly. However, this activity is influenced by the surface nature of the substrates. Compared to the Al_2_O_3_ substrate, the photodegradation on the AAO substrate took 80 minutes to reach the same level of degradation shown in Figure 5c. This slow degradation arises due to its hydrophobic nature, where more molecules concentrate on a single point and take more time.^[^ ^78^ ^]^ Consequently, an increased number of molecules gather on these surfaces, leading to an enhancement in SERS sensitivity. However, these substrates suffer from photodegradation in comparison to hydrophilic substrates.^[^ ^44^, ^79^ ^]^ Thus, our result reveals that hydrophilic surfaces are more sensitive than hydrophobic surfaces to photodegradation. The photodegradation rate is higher for the Al_2_O_3_ (84%) substrate than the AAO nanotemplate (79%), as shown in Figure 5d. Table S5 (Supporting Information) displays a comparison of the photodegradation times of different molecules through different substrates, confirming the good performance of our substrate. Overall, the improved photocatalytic efficiency is likely a result of the heterojunction formation, which facilitates the effective transfer of photogenerated charge and can decompose MB into smaller inorganic products.^[^ ^80^, ^81^ ^]^

### 2.4 Detection of Heavy Metal Ions (Hg^2+^)

Beyond commonly used probe molecules such as MB and R6G, F_4_TCNQ/MoS_2_/AAO demonstrate sensitive detection capabilities from a practical application point of view. The novel SERS substrate shows high sensitivity in detecting water contamination, which is of particular concern to the environment and human lives. Mercury ion (Hg^2+^) in the water environment is a major global environmental issue because of its high toxicity. It has the ability to accumulate in marine organisms and can eventually become a health hazard to humans when consuming seafood. The ions in solution are typically not identifiable directly using the Raman technique due to the small Raman scattering cross‐section area; hence, a method using “reporter molecules” is commonly employed. The sensitivity of the substrate increases as reporter molecules are attached to the ion structure.^[^ ^82^, ^83^ ^]^ Here, we used 4‐ATP as a reporter molecule with different concentrations due to the thiol functional group (‐SH), which has a high affinity for Hg^2+^. 4‐ATP contains a thiol (‐SH) functional group,^[^ ^84^ ^]^ which is crucial for its interaction with metal ions, particularly Hg^2+^, and its application in SERS‐based sensing. Further, the thiol group of 4‐ATP has a high affinity for Hg^2+^ ions. The sulfur atom in the thiol group can form a strong covalent bond with the mercury ions, resulting in the adsorption of Hg^2+^ onto the 4‐ATP molecules. This binding event induces conformational changes in the 4‐ATP molecule.^[^ ^85^, ^86^ ^]^ Further, positively charged Hg^2^^+^ ions are attracted to the negatively charged sulfur atoms on the MoS_2_ surface due to electrostatic forces and could strongly bind to Hg^2+^.^[^ ^87^, ^88^ ^]^ Moreover, the AAO nanocavity structure can act as a vessel for Hg^2+^ ions, increasing their local concentration at the F_4_TCNQ/MoS_2_ interface, which enhances the probability of interaction and detection.^[^ ^89^ ^]^ Owing to all these key aspects of our material, which support a good fit to detect Hg^2+^.

**Figure 6:**
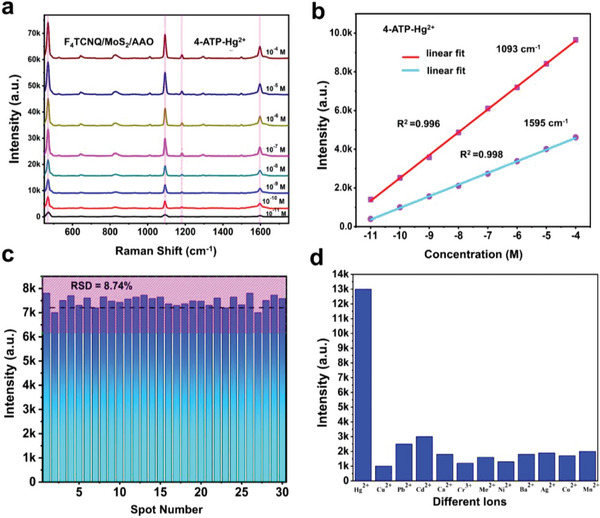
a) SERS of 4‐ATP and Hg^2+^ on F_4_TCNQ/MoS_2_/AAO with different concentrations. b) Linear fitting R^2^ of 1093 cm^−1^ and 1595 cm^−1^ of 4‐ATP‐Hg^2+^. c) RSD of Hg^2+^ on F_4_TCNQ/MoS_2_/AAO. d) selectivity of different metal ions.

After dropping 4‐ATP on the F_4_TCNQ/MoS_2_/AAO heterostructure, it adsorbs a sufficient amount of reporter molecule on its surface and exhibits high sensitivity to Hg^2+^ in water. The functionalized surface was immersed in an Hg^2+^ aqueous solution for 30 min, reporting a gradual increase with an increase in concentration [10^−4^ to 10^−11^ M] as illustrated in **Figure** **6**a. The signal of 4‐ATP at 1079 cm^−1^ is chosen as the internal standard in our experiment to correct deviations and create calibration curves. This choice is based on its location in a spectral band without interference and its response intensity. Small 4‐ATP molecules can significantly reduce their impact on certain reactions with metal ions compared to other large molecules. Furthermore, the surface of the heterostructure is covered with a large number of very small 4‐ATP molecules, thereby preventing the nonspecific absorption of interfering substances like inorganic chemicals, DNA, proteins, bacteria, etc., in practical scenarios.^[^ ^90^ ^]^ The correlation between spectral intensity and the concentration of Hg^2+^ is illustrated in Figure 6b, with R^2^ approximately reaching 0.996 and 0.998 for 1093 cm^−1^ and 1595 cm^−1^, respectively. In order to evaluate the consistency of the system, a total of 30 randomly selected points were examined. The obtained results, as depicted in Figure 6c, indicate that the RSD is below 9%. This finding demonstrates a high level of repeatability for the proposed SERS sensor. To confirm the selectivity of Hg^2+^ to the SERS platform, the sensor was tested for interference from additional metal ions, including Cu^2+^, Pb^2+^, Cd^2+^, Ca^2+^, Cr^3+^, Mg^2+^, Ni^2+^, Ba^2+^, Ag^2+^, Co^2+^, and Mn^2+^, under the same conditions. Compared to other metal ions, the SERS response to Hg^2+^ demonstrated unparalleled selectivity (Figure 6d). The system performed better with all metal ions, showing less interference from coexisting ions on the SERS platform. Figure 6b shows that only Hg^2+^ significantly alters the SERS signal of 4‐ATP and creates a new peak at 482 cm^−1^, demonstrating excellent selectivity of the SERS sensor.

## 3 Conclusion

By combining a large surface area and high‐quality CVD‐grown MoS_2_ followed by the thermal deposition of F_4_TCNQ on AAO, we obtained an ultrasensitive F_4_TCNQ/MoS_2_/AAO based on CM and EM enhancement. A comparison of F_4_TCNQ/MoS_2_ has been carried out on three AAO (400, 900, and 1500 nm) and one flat Al_2_O_3_ substrate, revealing that AAO with a depth of 1500 nm has remarkable Raman enhancement. However, the uniform and controlled growth of nanocavities is difficult. The heterostructure F_4_TCNQ/MoS_2_ improves charge transfer through a photo‐induced charge transfer mechanism from the Raman probe. By introducing the AAO nanotemplate, the EM enhancement is strongly coupled with the molecular and CT resonances, leading to efficiently enhanced SERS spectra. The underlying mechanisms behind improved SERS performance were evaluated through UPS (DOS near the Fermi level) and COMSOL (electric field distribution). The overall observed enhancement arises from both CM and EM, which show an ultralow LOD (9.0×10^−16^ M) and high EF, comparable to or even superior to noble metal‐based substrates. More importantly, the as‐prepared substrate exhibits stability for up to 3 months and shows rapid response, even in seconds, after dropping the probe molecule. Taking advantage of the organic/inorganic heterostructure for the photodegradation of MB. The observed degradation of a hydrophilic surface is faster than that of a hydrophobic surface due to fewer atom accumulations compared to a hydrophobic surface. Finally, the innovation in the SERS performance is the detection of Hg^2+^ ions in pure water with a low LOD of 10^−11^ M. Our results suggest that nanoarchitectured templates and organic/inorganic heterostructures can be promising candidates in different sensing areas, especially in biological molecules, because their large size can be easily adjusted in such nanocavities.

## 4 Experimental Section

### Materials and Regents

The V‐shaped AAO (450–100–1500 nm) nanotemplates with a nanocavity diameter of 450 nm, a distance between nanocavities of 100 nm, and different depths of (400, 900, and 1500 nm) were purchased from Shenzhen Top Membranes Technology Co., Ltd. Methylene Blue and Rhodamine 6G were purchased from Sinopharm Chemical Reagent Co., Ltd. The 4‐aminothiophenol (C6H7NS, GC), ammonium tetrathiomolybdate (H8MoN2S4, 99.95%) have been obtained from Shanghai Aladdin Biochemical Technology Co., Ltd. The Hg^2+^, Cu^2+^, Pb^2+^, Cd^2+^, Ca^2+^, Cr^3+^, Mg^2+^, Ni^2+^, Ba^2+^, Ag^2+^, Co^2+^, and Mn^2+^ solutions were received from Tongroma Technology (Beijing) Co., Ltd.

### Synthesis of the MoS_2_ and F_4_TCNQ/MoS_2_

Four substrates were chosen, including three AAO with different depths (400, 900, and 1500 nm) and one Al_2_O_3_ substrate for SERS comparison. The F_4_TCNQ/MoS_2_ heterostructure was synthesized by a two‐step process, according to the literature.^[^ ^30^ ^]^ The (NH_4_)_2_MoS_4_ powder (0.0120 g) with 99.95% purity was dissolved in ethylene glycol (1 mL) to make a mixed solution. This solution was spin‐coated on AAO and Al_2_O_3_ substrates at 800 rpm min^−1^ for 5 s followed by 3000 rpm min^−1^ for 15 s. The synthesis procedure involved placing a boat at the center of the furnace containing Al_2_O_3_ and AAO substrates, which were pre‐spin‐coated to form a uniform film. 100 sccm of H_2_ gas was used as the carrier gas in the entire process at a temperature of 550 °C for three hours. Thus, a few layers of MoS_2_ were synthesized by the chemical vapor deposition process in the first step. In general, the process of fabricating organic/inorganic heterostructure involves two steps involving the deposition of less stable organic elements onto pre‐synthesized (more stable inorganic materials). Thus, in the second step, F_4_TCNQ organic powder was placed in a quartz boat covered by pre‐synthesized 1 cm × 1 cm MoS_2_/AAO with direct deposition of F_4_TCNQ by thermal evaporation, leading to the growth of disconnected nanoparticles on the MoS_2_ surface. The growth temperature rate for enabling F_4_TCNQ deposition on the MoS_2_/AAO and MoS_2_/Al_2_O_3_ substrates was 5 °C per minute after reaching a temperature of 100 °C for 15 min.

### Characterization

The morphology of grown samples was analyzed using a scanning electron microscope (SEM, Zeiss Sigma 500) and elemental mapping analyses with field emission SEM (FESEM, Hitachi, Regulus 8100). Crystallinity analysis was performed by XRD (Bruker, D8‐Advance) Cu Kalpha. Raman, photoluminescence (PL), and SERS performance were obtained by using the Horiba LabRam HR Evolution system, using 325 nm and 532 nm excitation wavelengths with a laser spot size of ≈2 microns, respectively. Ultraviolet‐visible (UV‐vis) absorption measurements were carried out by employing a double‐beam UV‐vis spectrometer (Shimadzu, SolidSpec‐3700iDUV). The surface elemental chemical states were analyzed by X‐ray photoelectron spectroscopy (XPS) through Thermo Fisher Scientific, Escalab 250Xi. The valence band states of the synthesized materials were investigated by employing UPS through the PHI5000 VersaProbe III (Scanning ESCA Microprobe).

### Simulation Details

Using COMSOL simulation, the EM enhancement within nanocavities of AAO templates with different depths was simulated and evaluated using SEM results. An incident laser wavelength of 532 nm was irradiated onto the AAO surface in the Z direction using the electromagnetic wave frequency domain. Periodic boundary conditions were applied to the x and y‐axes with a perfect matching layer condition along the z‐axis. The F_4_TCNQ/MoS_2_/AAO was simplified to a hollow V‐shaped cone with a certain thickness. The three AAOs with fixed top and bottom diameters (450, 100 nm) and different depths of 400 nm, 900 nm, and 1500 nm were simulated. The thickness of the few MoS_2_ layers is 2.12 nm, and the size of the F_4_TCNQ is set to be 38 nm with an average gap distance of ≈108 nm. The effects of EM enhancement inside bare AAO, MoS_2_/AAO, and F_4_TCNQ/MoS_2_/AAO were compared.

### SERS Measurement

MB and R6G were dissolved in water to form solutions with different concentrations. Subsequently, a series of solution concentrations ranging from 5×10^−5^ to 5×10^−16^ M were prepared through sequential dilution processes. The Raman spectra were acquired using an ×50x objective lens with a numerical aperture of 0.75. For SERS measurements, a 532 nm wavelength laser served as the excitation source. The laser spot had a diameter of approximately 1 µm, and the spectral resolution was ≈1 cm^−1^. To prevent structural changes induced by laser heating, the laser power was set at 0.1 mW. A 1800 lines mm^−1^ diffraction grating with a focal length of 250 mm was employed. To evaluate the SERS performance, 50 µL of different probe solutions were dropped on F_4_TCNQ/MoS_2_/AAO and dried in the air. The stability of the sample was investigated by measuring the SERS signal at 10^−5^ M of MB. To evaluate reproducibility, five batches of F_4_TCNQ/MoS_2_/AAO were chosen under the same experimental conditions, with each sample contributing five spectra.

### SERS of Hg^2+^ Ions in Water

For practical implications, an aqueous solution of heavy metal ions (Hg^2+^) was used to detect water contamination. For the Hg^2+^ SERS detection, aminothiophenol (4‐ATP) was chosen as a Raman reporter molecule to make different concentrations of Hg^2+^. A solution of 4‐ATP was prepared by dissolving 0.012519 g of 4‐ATP into ethanol to get a stock solution of 10^−2^ M.^[^ ^91^ ^]^ Next, a range from 10^−4^ to 10^−11^ M was obtained through serial dilution method. The successful loading of 4‐ATP was carried out by immersing F_4_TCNQ/MoS_2_/AAO substrate in 4‐ATP solution for 30 min. After being picked out from the solution, the substrates were rinsed with deionized water for three times to remove the non‐specifically attached 4‐ATP molecules from the SERS substrates and dried. A stock solution of Hg^2+^ (stored at 4 °C) is mixed with a reporter molecule with a concentration ranging from 10^−4^ to 10^−11^ M. 50 µL of mixed solution adsorbs on F_4_TCNQ/MoS_2_/AAO for evaluating SERS performance.

### Photocatalytic Degradation Experiment

The F_4_TCNQ/MoS_2_ composite was evaluated for its photocatalytic activity by removing MB dye using a Xenon lamp as a source light operating at 30 V. For our setup, the Xenon lamp is calibrated to provide a power flux density of ≈200 mW cm^−2^ with a distance from the light source to the substrate kept at 4 cm. The light was turned on at different time intervals. The rate of degradation for MB was determined by the following equation:

(11) $$\tau =\frac{A_{o}-A_{\tau }}{A_{o}}\;\times 100\%$$

where τ is the photodegradation rate, while *A_o_* and *A*_τ_ represent the initial and final peak intensity after UV light exposure, respectively.

## Conflict of Interest

The authors declare no conflict of interest.

## Supporting information



Supporting Information

## Data Availability

The data that support the findings of this study are available in the supplementary material of this article.
