# Supplementary material for: V‐Shaped Heterostructure Nanocavities Array with CM and EM Coupled Enhancement for Ultra‐Sensitive SERS Substrate
Source: Adv Sci (Weinh). 2024 Oct 28;11(48):2409838. doi: 10.1002/advs.202409838 (PMC11672317; doi:10.1002/advs.202409838)
Supplement: Supplementary file 1 — Supporting Information [file ADVS-11-2409838-s001.docx]

**Supporting information**

**V-shaped Heterostructure Nanocavities Array with CM and EM Coupled Enhancement for Ultra-sensitive SERS Substrate**

*Abdur Rahim, Liqi Ma, Muhammad Saleem, Baiju Lyu, Muhammad Shafi, Yuxin You, Mingyue Li, Xiaoyu Zhang, Mei Liu^*^*

*School of Physics and Electronics, Shandong Normal University, Jinan, 250038, China*

*^*^Corresponding author:* [liumei@sdnu.edu.cn](mailto:liumei@sdnu.edu.cn)


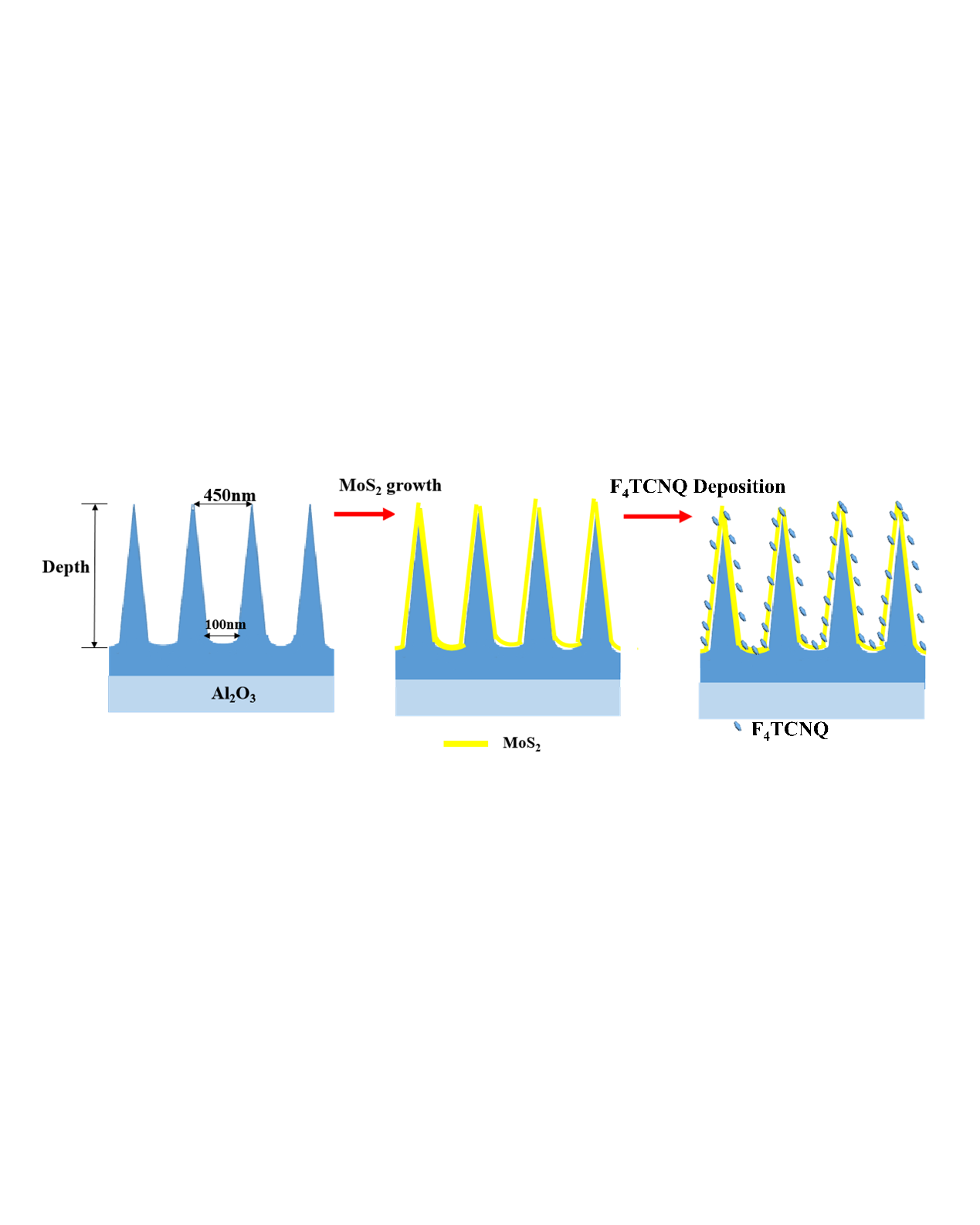


**Figure S1.** Schematic illustration of the growth of MoS_2_ and F_4_TCNQ on AAO.

**
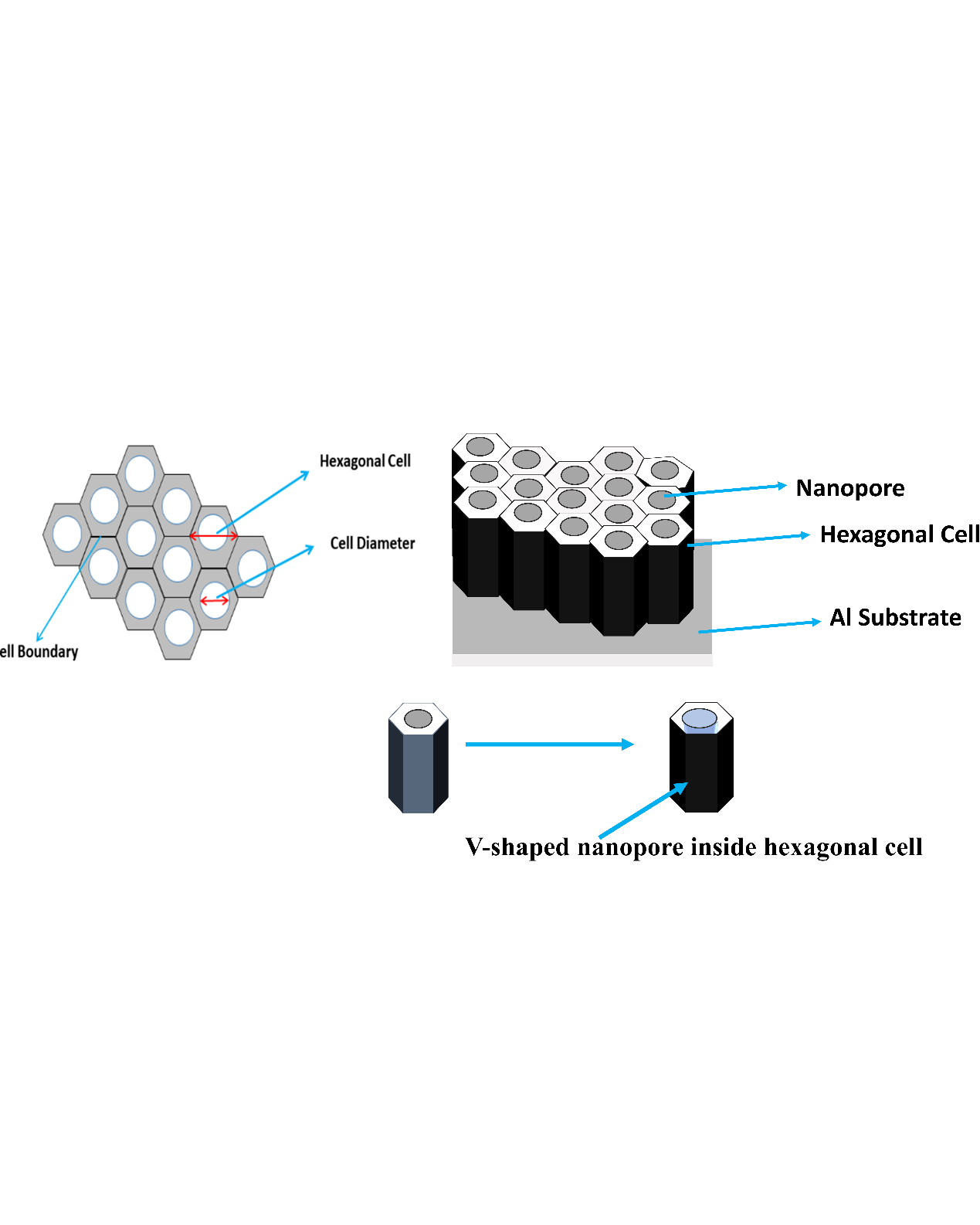
**

**Figure S2.** Geometrical interpretation of the AAO model.

The AAO template offers numerous appealing advantages, including nanoscale channels, customizable pore dimensions, and lengths, ordered arrays of pores, and a substantial surface area. AAO membranes are predominantly employed as templates for the controlled growth of diverse nanostructures, such as nanotubes, nanorods, and nanowires, using a variety of organic and inorganic materials ^[1]^. Furthermore, the mechanical, chemical, and physical properties of materials can be effectively modified by utilizing the AAO template approach to adjust their size, shape, and structure. Their extraordinary properties, coupled with tunability, render AAO membranes highly suitable for a myriad of applications. The porous AAO template's structure resembles a densely packed hexagonal arrangement of parallel cylindrical nanochannels, akin to honeycombs, with diameters ranging from 10 to 450 nm ^[1]^. The AAO template consists of three layers: An aluminum base layer, an AAO bottom layer, and an effective AAO layer. The effective layer is the top layer containing V-shaped nanocavities arranged in honeycomb order shown in Figure S2.

**Figure S3.** SEM image of F_4_TCNQ nanoparticles distributed on MoS_2_/Al_2_O_3._


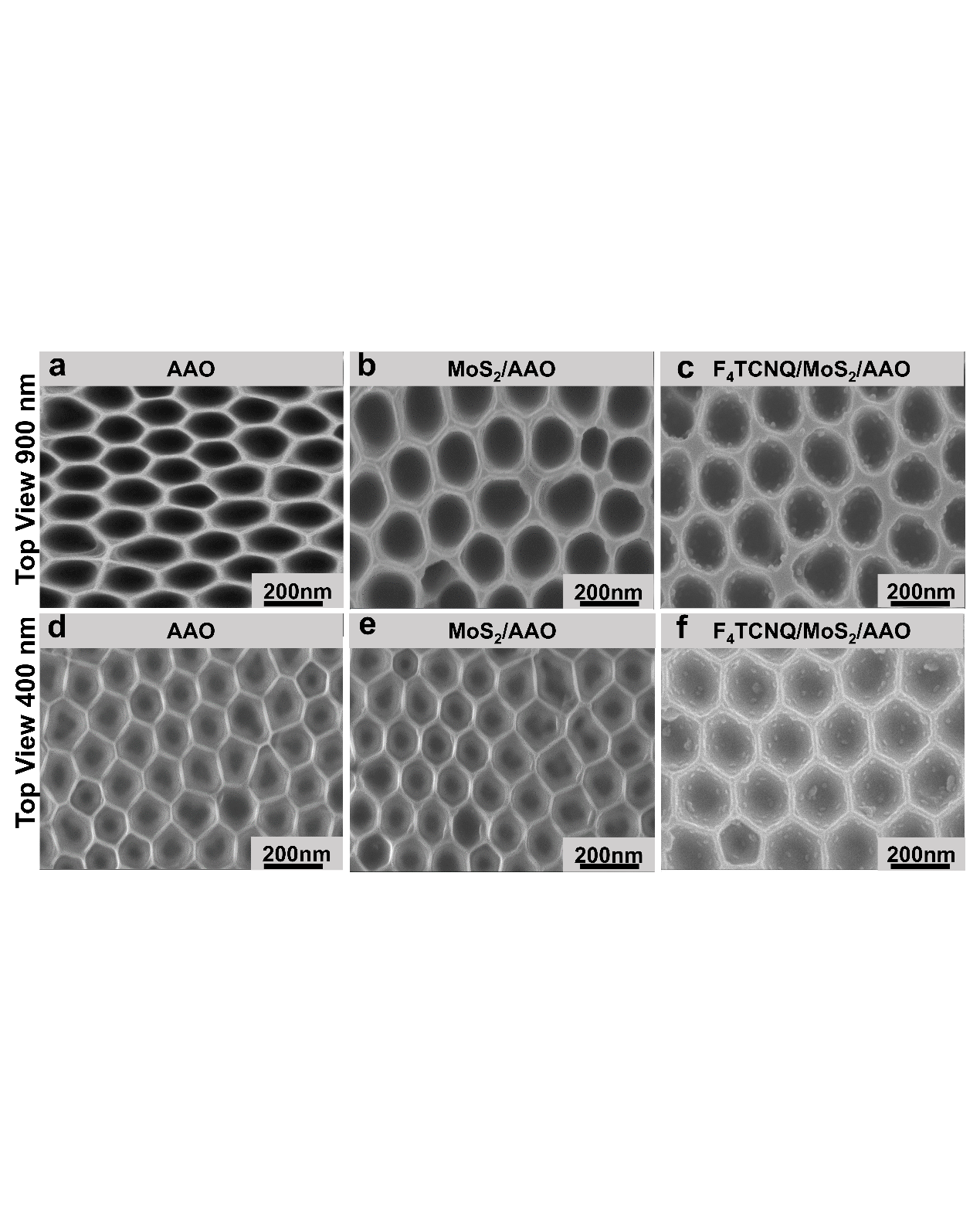


**Figure S 4.** Top view of the AAO with different depths. (a) bare AAO (900 nm). (b) MoS_2_ deposited AAO (900 nm). (c) F_4_TCNQ deposited on MoS_2_/AAO (900 nm). (d) bare AAO (400 nm). (e) MoS_2_ deposited AAO (400 nm). (f) F_4_TCNQ deposited on MoS_2_/AAO (400 nm).

The bottom of the 400 nm AAO model can be seen more clearly among other AAO models. The top view diameters were found to be about 450 nm on average for all three AAO templates. To measure the depth of the V channel and the bottom diameter of the nanopore, the AAO substrate was broken at the midpoint and captured the morphology of the cross-sectional view. As depicted in Figure S5, a consistent structure is observed through each template with varying depths (~1500, 900, 400 nm) and a nearly fixed bottom diameter around 100 nm.

**The final geometry of the nanocavities**

The height and diameter of pristine nanocones will determine the starting point for the coating process. The shape and diameter are slightly changed according to MoS_2_ coating. The grown MoS_2_ is trilayer according to our Raman analysis possess thickness of approximately 2 nm coated on the AAO nanocones surface. This will reduce the top and bottom diameter to approximately 96 nm (Initial 100 nm) and 446 nm (Initial 450 nm), respectively while the depth decreases by 2nm. So, the final dimension reduces and geometry of the AAO change from hexagonal to circular shape. The F_4_TCNQ nanoparticles are not like MoS_2_ layers which also affect the geometry but not at each and every point of nanocones because these are disconnected particles.


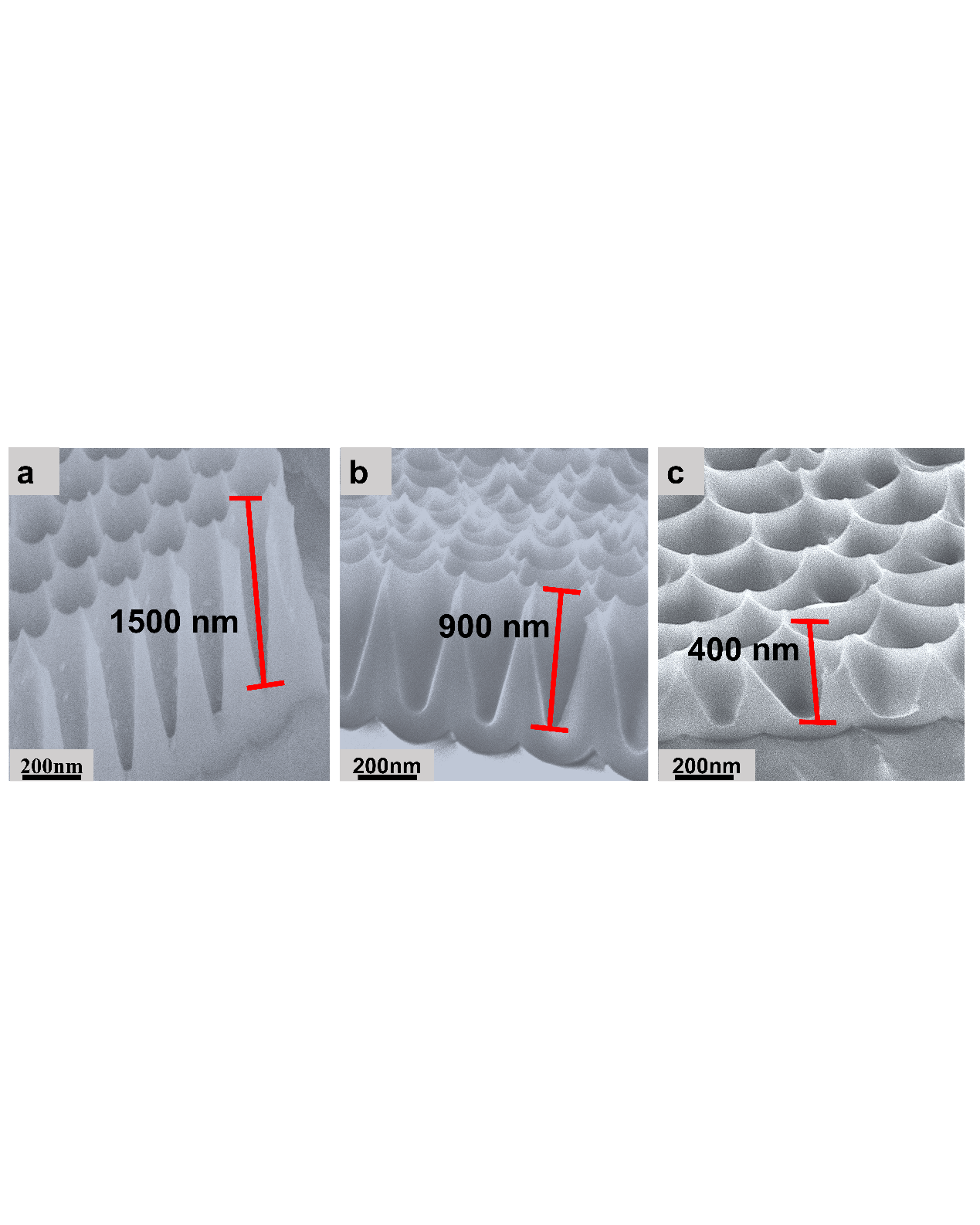


**Figure S5.** Side view of three AAOs with different depths. (a) 1500 nm AAO. (b) 900 nm AAO. (c) 400 nm AAO.

**About uniformity of MoS_2_ and F_4_TCNQ**

The precursor solution for MoS_2_ is spin-coated with high rpm speed to get a uniform film and for F_4_TCNQ the as-prepared MoS_2_ kept close to the organic powder to ensure more particles can reach the substrate through physical vapor deposition led to almost homogenously dispersed nanoparticles. The distribution of MoS_2_ is to be uniform inside the nanocavity and on the top of AAO because we obtained the same frequency difference (23 cm^-1^) between two modes of MoS_2_ A_1g_ and E^1^_2g_ which indicates MoS_2_ trilayer. Further, this finding, supported by additional EDS mapping that showed a consistent distribution of Mo and S across the nanocavity (Figure S6), indicates a homogenous dispersion of MoS₂ within the AAO template. The content of MoS_2_ is highlighted with Mo (dark orange color) and S (dark green color). Moreover, the distribution of F_4_TCNQ is likely to be uniform as confirmed by EDS mapping but not as MoS_2_ displayed in Figure S6(d-f) because these particles are disconnected. The particles distributions are almost homogenous inside the nanocavity revealed through the SEM image as shown in Figure S6.

**
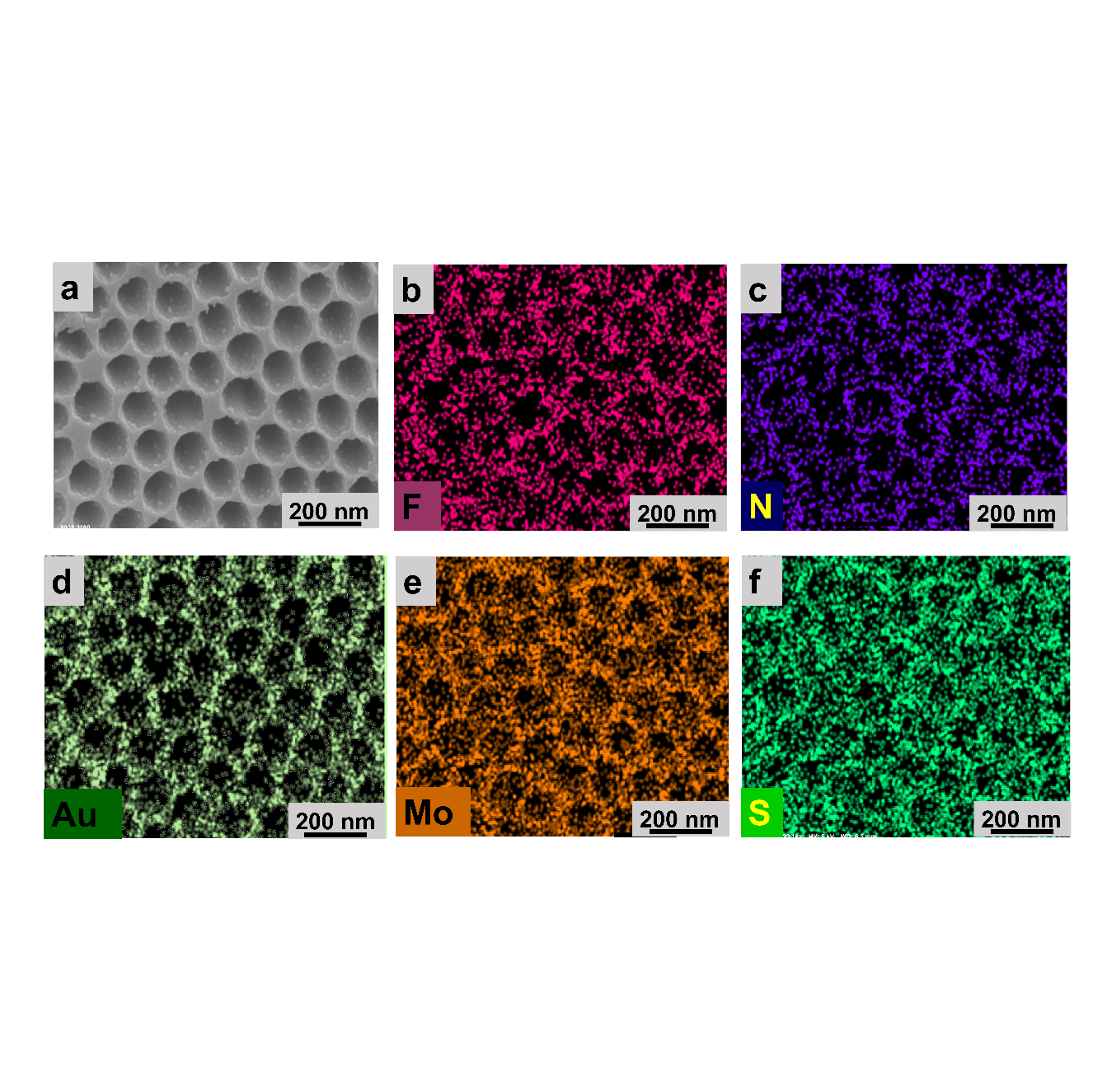
**

**Figure S6.** Energy-dispersive spectrometer (EDS) elemental mapping of F_4_TCNQ/MoS_2_ grown on AAO (a-F).

**(b)**


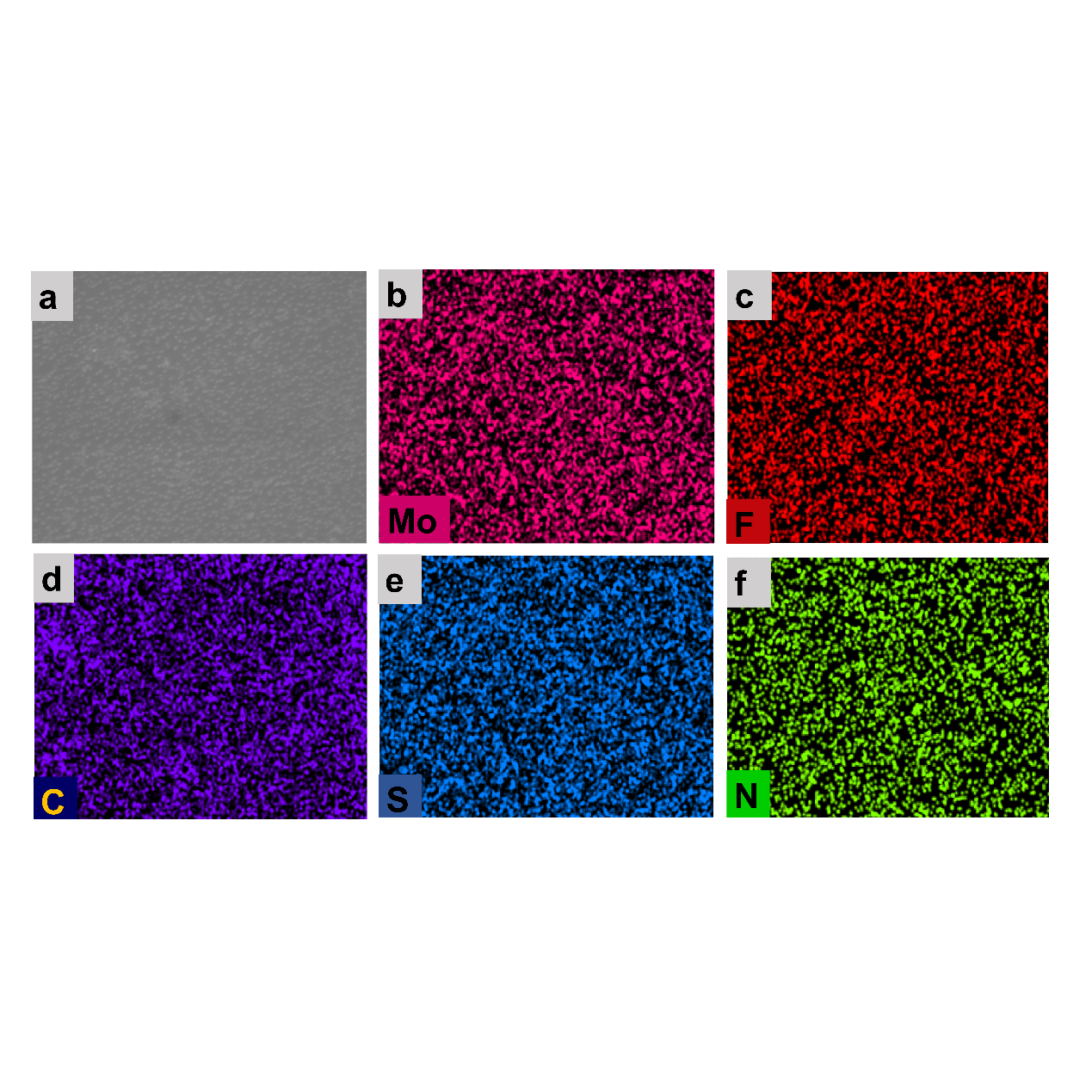


**Figure S7.** EDS mapping of F_4_TCNQ/MoS_2_ on Al_2_O_3_ substrate.

*
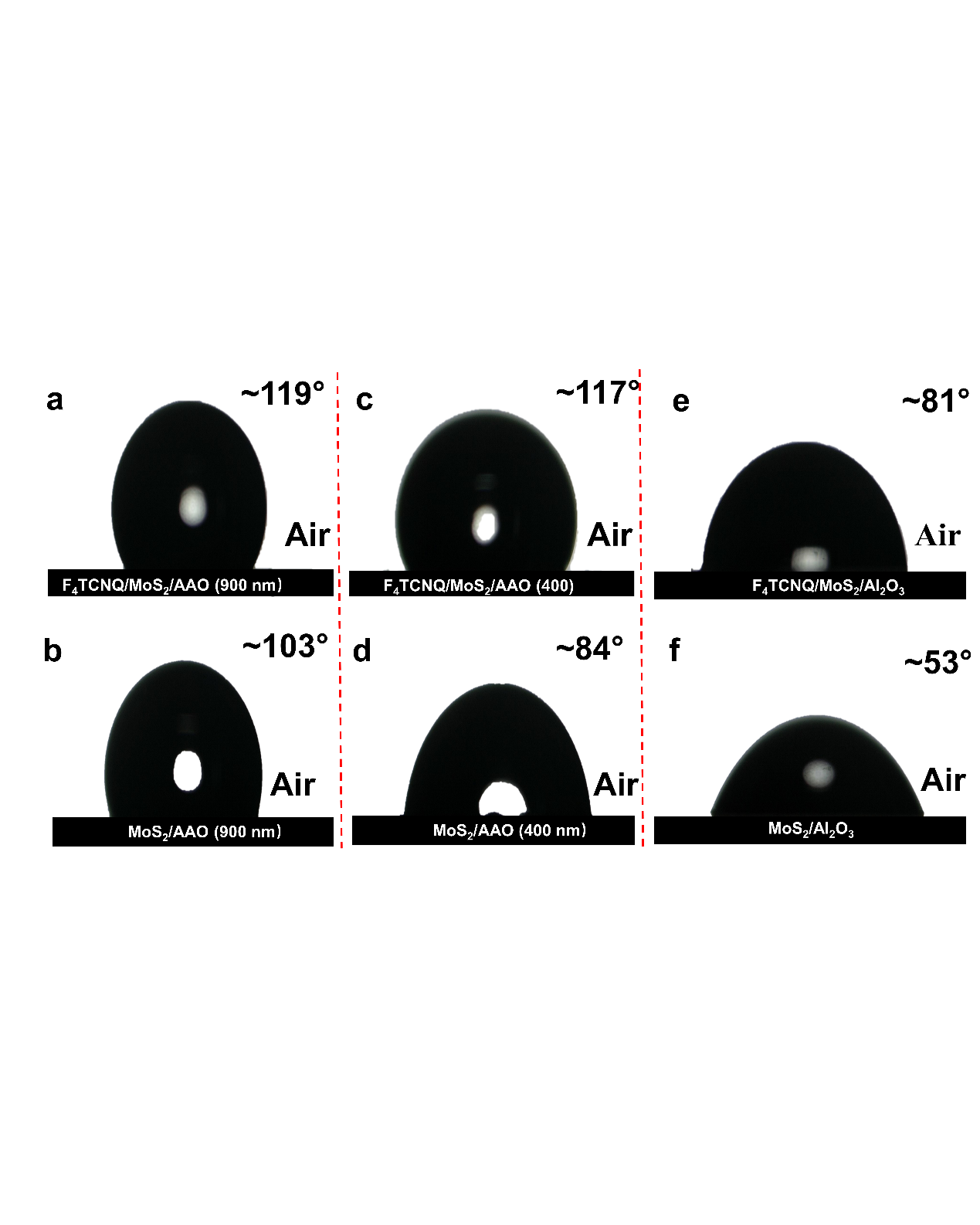
*

**Figure S8.** Optical images of the contact angle (CA) of water droplets on different substrates. (a-d) CA of F_4_TCNQ/MoS_2_ and MoS_2_ grown on AAO (900 nm, 400 nm), respectively. (e-d) CA of F_4_TCNQ/MoS_2_ and MoS_2_ on Al_2_O_3._

**
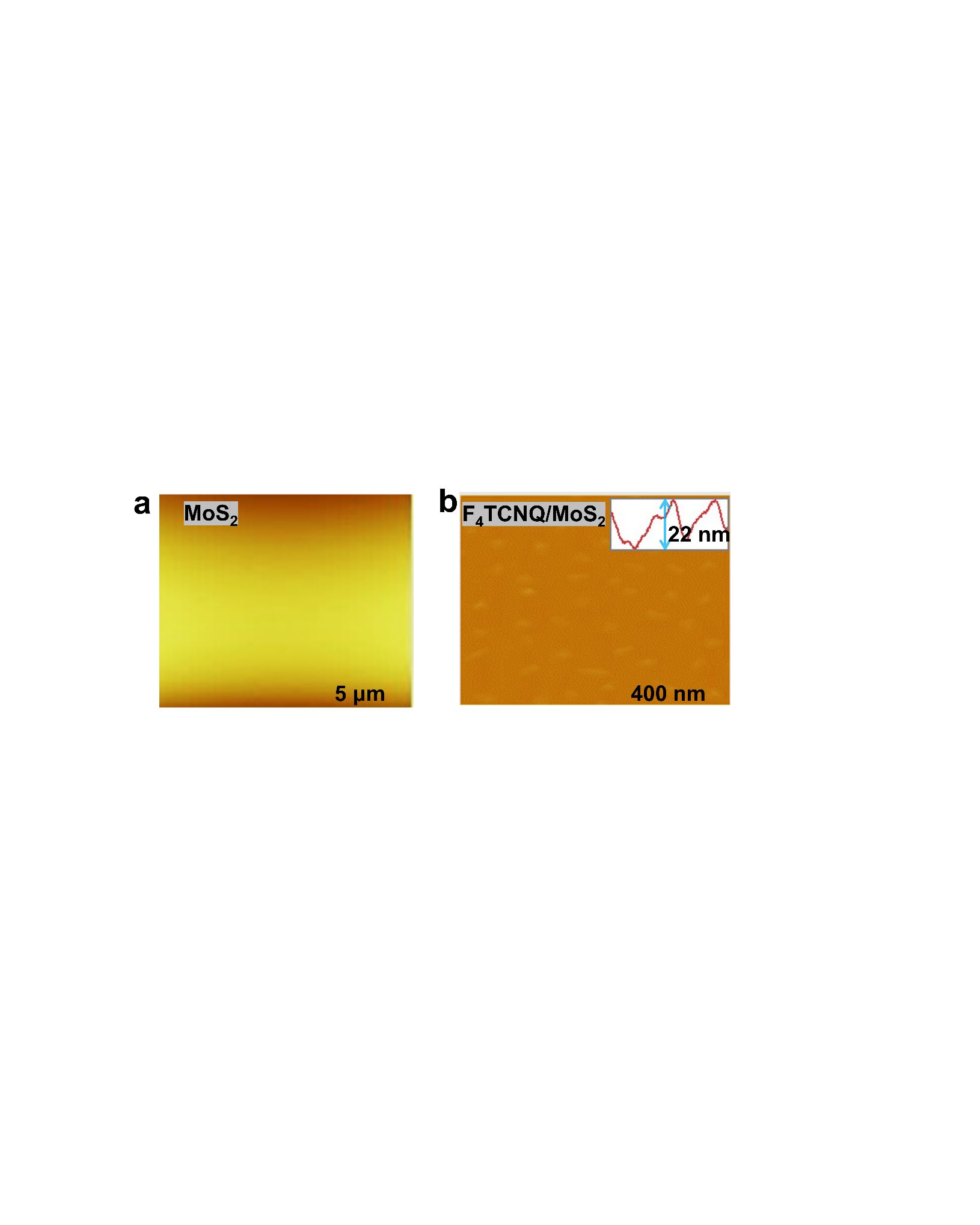
**

**Figure S9.** AFM analysis of (a) MoS_2_ and (b) F_4_TCNQ/MoS_2_.

The AFM result is obtained for the substrate Al_2_O_3_ because the AAO surface is comparatively rough and affects the performance of the cantilever of the AFM. Figure S9a and b display the surface topography of 5 µm × 5 µm and 400 nm × 400 nm, with an evenly distributed surface of MoS_2_ and disconnected island-like F_4_TCNQ particles on MoS_2_, respectively, with an average height profile of approximately 20 nm.

**Figure S10.** Molecular geometry of Polymorph I view of F_4_TCNQ with no coplanar arranged molecules.

**Figure S11.** Raman peaks of MoS_2_ on different substrates.


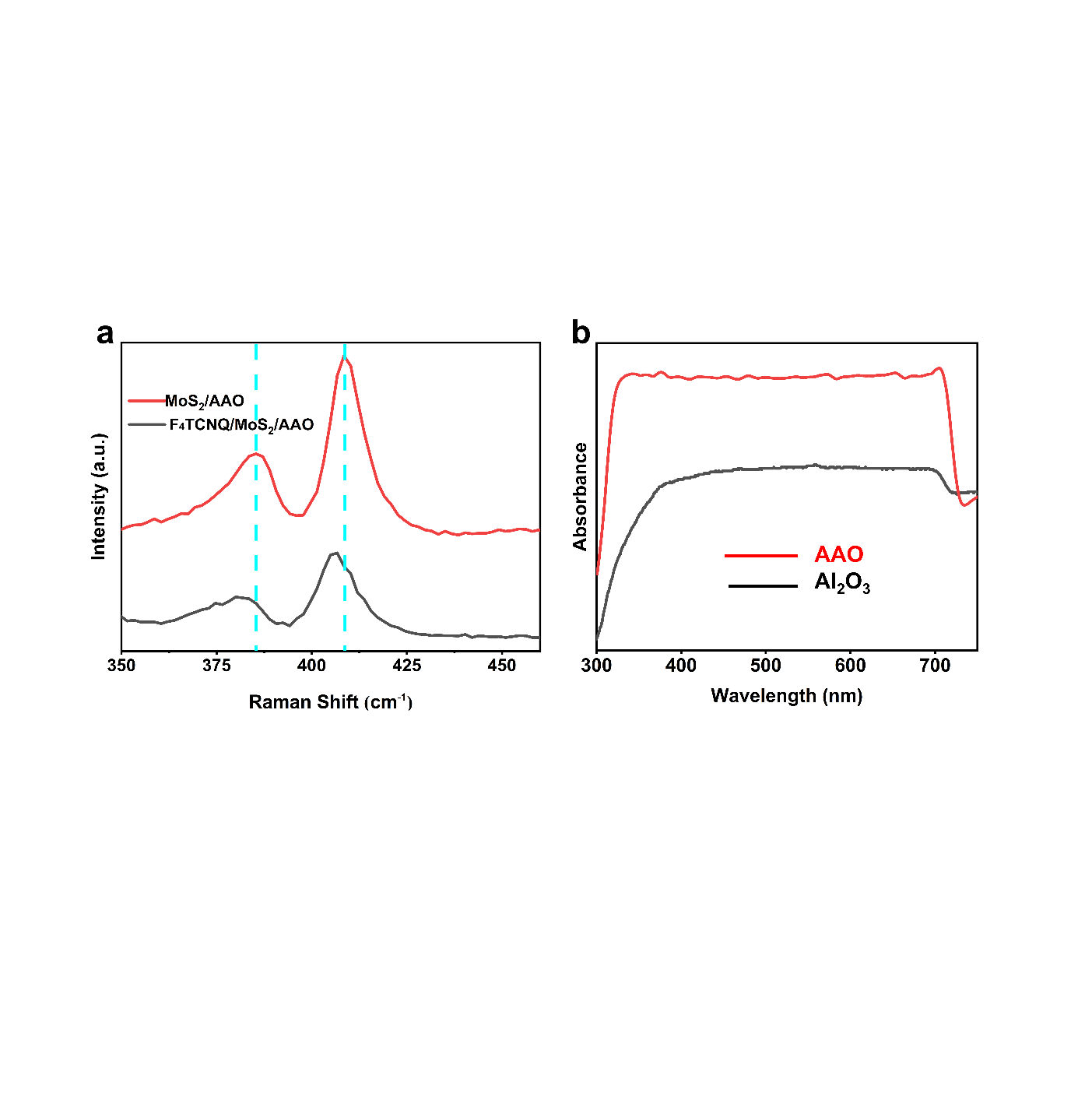


**Figure S12.** (a) Raman spectra of MoS_2_ and F_4_TCNQ/MoS_2._ (b) UV-Vis absorption spectra of AAO and Al_2_O_3._


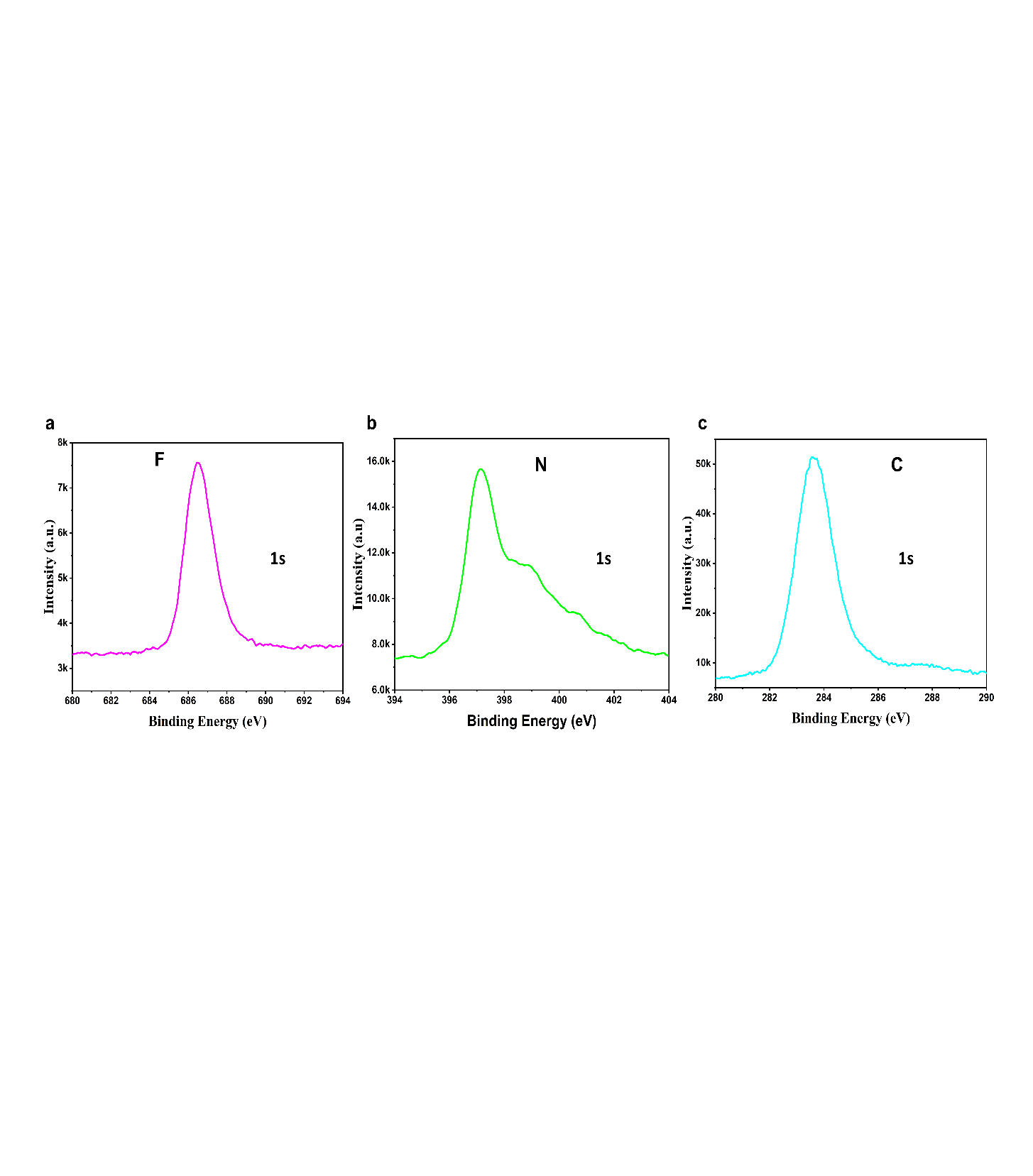


**Figure S13.** X-ray Photo-electron spectra of F, N, and C, respectively.

**Figure S14**. MB characteristic peak 1629 cm^-1^ on four different bare substrates.


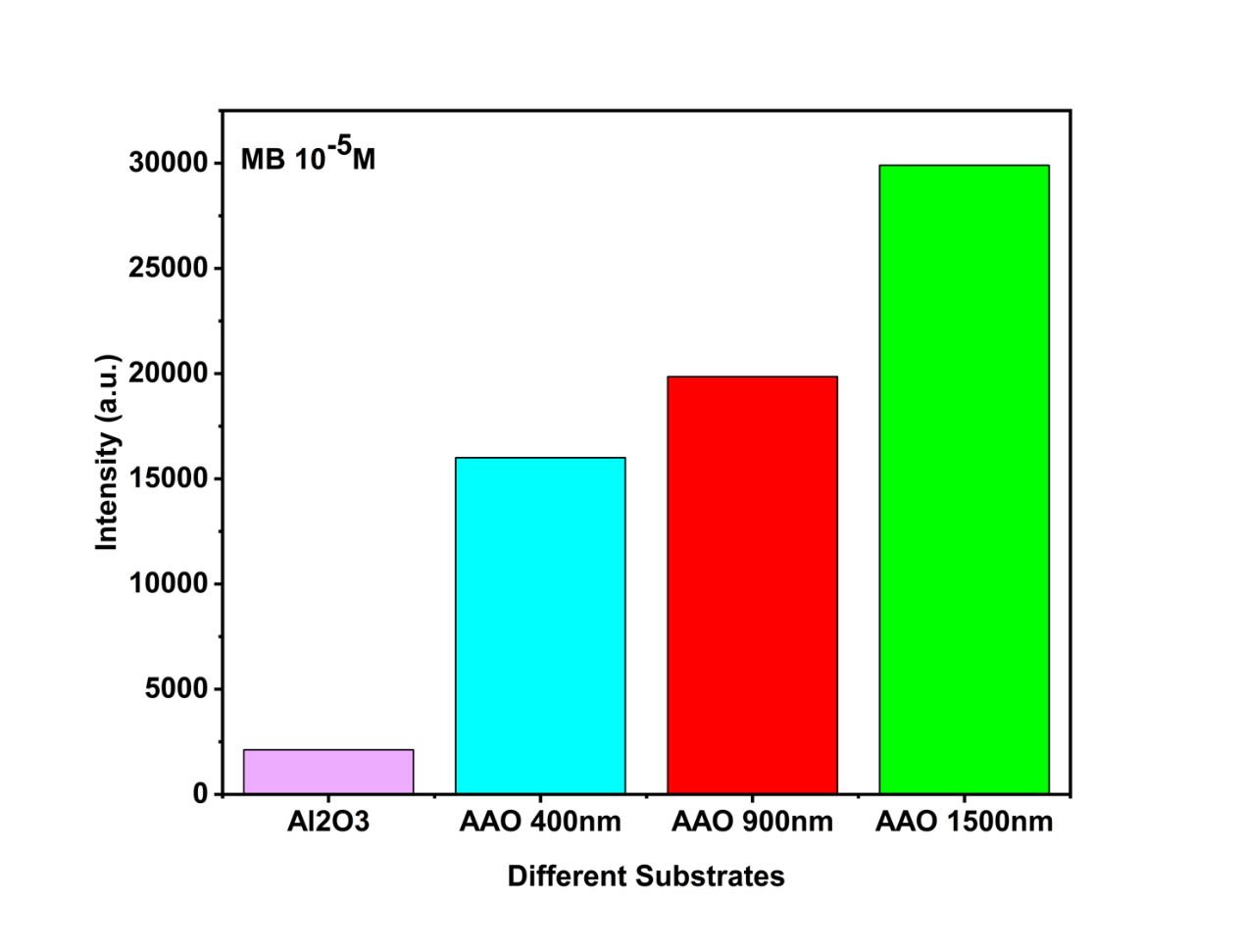


**Figure S15.** Comparison of Raman signal intensities at 1629 cm^-1^ of different substrates.

**Figure S16.** COMSOL simulation of MoS_2_/AAO and bare AAO.

To assess the electromagnetic enhancement attributed to nanopores of the AAO nanotemplate, COMSOL simulation was used. In the process of fabricating the SERS substrate, COMSOL analysis was used to computationally model and simulate the electric field distribution and strength of the F_4_TCNQ/MoS_2_ inside the AAO SERS substrates with different nanopore depths and fixed bottom and top diameters, as earlier discussed. The nanopore depths of 400 nm, 900 nm, and 1500 nm were simulated with monochromatic light of 532 nm placed 500 nm above the substrate. Periodic boundary conditions were set in both the X and Y directions, while perfectly matched layer boundary conditions were set on the Z-axis. As a result, the electromagnetic field distributions and intensities were collected and analyzed for the F_4_TCN/MoS_2_/AAO to find the best SERS substrate.

**Table S1.** Raman peaks band assignment of MB on different substrates.^[2]^

| MB Powder/cm^-1^ | AAO substrate/cm^-1^ | F_4_TCNQ/MoS_2_/AAO/cm^-1^ | Band Assignment |
| --- | --- | --- | --- |
| 1630 | 1623 | 1629 | ν(C-C) |
| 1405 | 1397 | 1399 | α(C-H) |
| 1304 | 1300 | 1303 | ν_symmetric_(C-N ) β(C-H )ν(C-C) |
| 1187 | 1184 | 1186 | ν(C-N) |
| 1152 | 1151 | 1155 | γ CH3α(C-H) |
| 1037 | 1041 |  | β(C-H) |
| 953 | 946 | 952 | γ CH3 α(C-H) |
|  | 861 | 864 | β(C-H)δ(C-H) |
| 769 | 769 | 771 | β (C-H) |
|  | 667 | 673 | γ(C-H) |
|  | 500 | 502 | δ(C-N-C) |
|  | 443 | 451 | δ(C-N-C) |

**Abbreviations:** s, strong; m, medium; w, weak; ν, stretching; α, in-plane deformation; β, in-plane bending; γ, out-plane bending; δ, skeletal deformation.

**SERS mapping:**

We have conducted additional SERS mapping experiments for MB at concentrations of 10^-15^ M and 10^-16^ M over a large area of 80 μm× 80 μm, with 4 μm spacing as shown in Figure S17. We successfully identified MB molecules (red color) within the laser-focused region. More red spots can be observed in the mapping due to the hydrophobic nature of our substrate, which does not allow the probe molecule to spread out as in the case of the hydrophilic substrates. The SERS spectra obtained from these specific regions clearly demonstrate the characteristic Raman peaks of MB, even at the extremely low concentrations of 10^-15^ M and 10^-16^ M

**
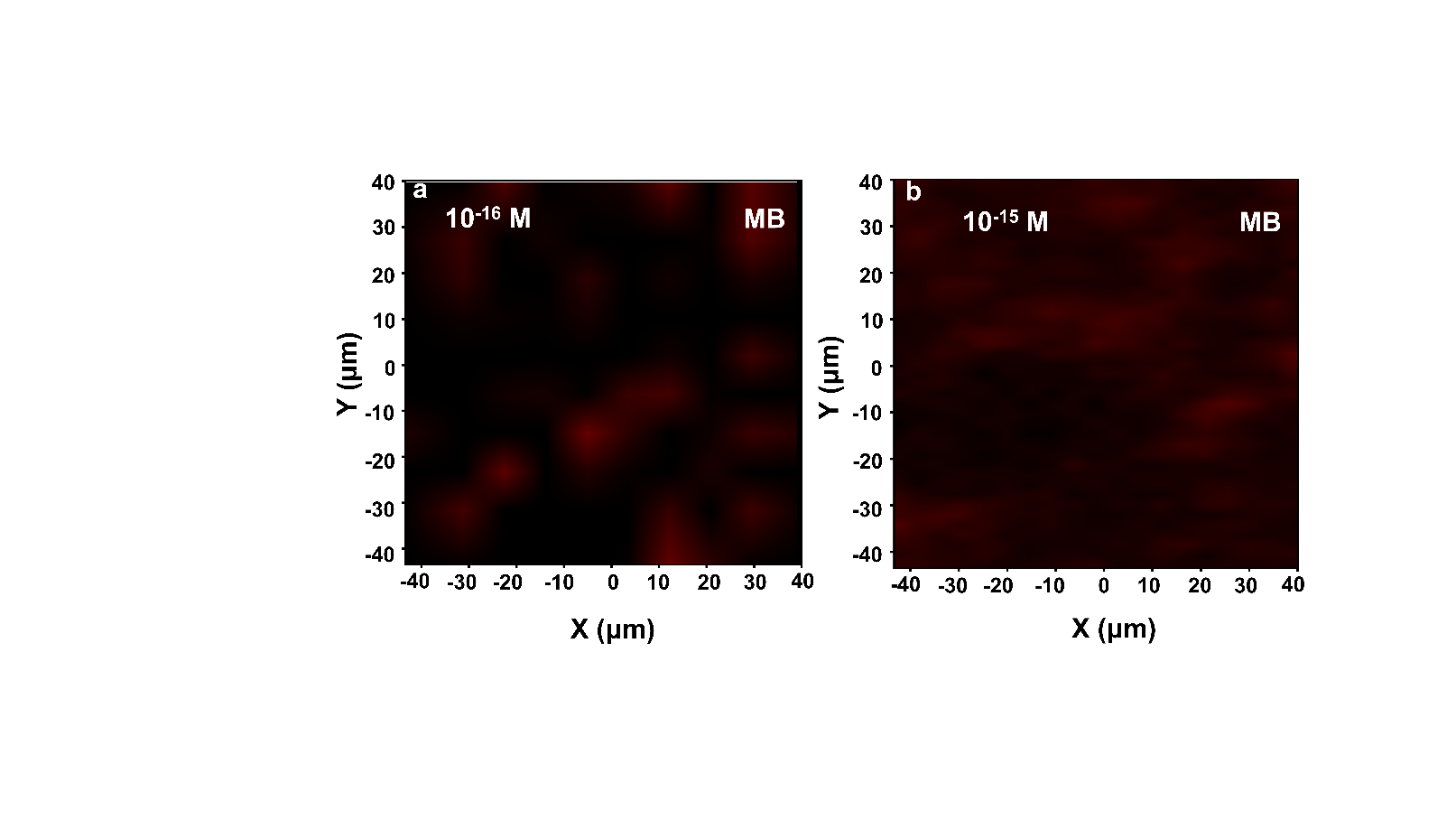
**

**Figure S17.** SERS mapping of MB peak (1629 cm^-1^) at different concentrations of (a) 10^-16^ M and (b) 10^-15^ M.

**Calculation of enhancement factor EF;**

The enhancement factor (EF) is a crucial metric in Surface-Enhanced Raman Scattering (SERS) analysis, providing insight into the amplification of Raman signals in the presence of a substrate. The formula to calculate EF is expressed as:

EF = $\frac{I_{\mathrm{SERS}}}{I_{\mathrm{bulk}}}\times\frac{N_{\mathrm{bulk}}}{N_{\mathrm{SERS}}}$

Here, $I_{\mathrm{SERS}}$ and $I_{\mathrm{bulk}}$ represent the intensities of a selected Raman peak in SERS and non-SERS spectra, respectively. $N_{\mathrm{SERS}}$ and $N_{\mathrm{bulk}}$ denote the average number of molecules in the scattering area for SERS and non-SERS measurements. The expressions for $N_{\mathrm{SERS}}$ and $N_{\mathrm{bulk}}$ are given bellow:​

$N_{\mathrm{SERS}}=\frac{\mathrm{CV}N_{A}A_{\mathrm{Raman}}}{A_{\mathrm{sub}}}$

​

$N_{\mathrm{bulk}}=\frac{\rho\mathrm{hN}_{A}A_{\mathrm{Raman}}}{M}$

​

The molar concentration of the analyte solution is denoted as C, while V represents the volume of the droplet. Additionally, N_A_ refers to the Avogadro constant. The laser beam's confocal depth (h) is set at 21 μm. The molecular mass M and density ρ of bulk MB is 373.9 g/mol and 1 g/cm³, respectively. The Raman scanning process involves a laser spot area A_Raman_ with a diameter of 2 μm. The effective area of the substrate A_Sub_ is determined by spreading a droplet of 20 mL volume on the substrate, resulting in a circle of 1cm^2^ area after solvent evaporation. To establish a non-SERS active reference, data for MB at a concentration of 10^-4^ M on a bare AAO substrate was used shown in Figure S17. The $I_{\mathrm{SERS}}$ and $I_{\mathrm{bulk}}$ of were collected about 28 and 31345 counts, respectively.

$EF=\frac{18}{3.1345\times{10}^{4}}\times\frac{\rho hA\mathrm{sub}}{C\mathrm{VM}}=\frac{18}{3.1345\times{10}^{4}}\times\frac{1.01\times21\times{10}^{-6}\times1\times{10}^{-4}}{1\times{10}^{-16}\times20\times{10}^{-6}\times373}=1.2\times{10}^{9}$

**Relative standard deviation (RSD)**

The RSD is a crucial metric for assessing the reproducibility and consistency of SERS signals across different substrate areas (included into supporting information after EF calculation).

RSD was calculated by the following equation:

$$RSD=\frac{\sqrt{\frac{\sum_{i=1}^{n} \left( I_{i}-\bar{I} \right)^{2}}{n-1}}}{\bar{I}}$$

Where $\mathbf{I}_{\mathbf{i}}$ is the SERS peak intensity collected from each point at 1629 cm^-1^, $\bar{I}$ represent the average of all SERS peak intensities, and n is the number of measured spectra which is 20 in this case. The collected data of Raman peaks intensities are 25120, 25811, 24923, 24502, 28410, 25253, 28316, 28512, 29037, 24670, 29721, 29509, 29031, 28533, 28038, 26576, 26135, 25566, 25149, and 29930. These high-intensity MB peaks were obtained at a concentration of 10^-5^ M. By putting all these values, the calculated RSD found to be 7.1% according to the above equation.


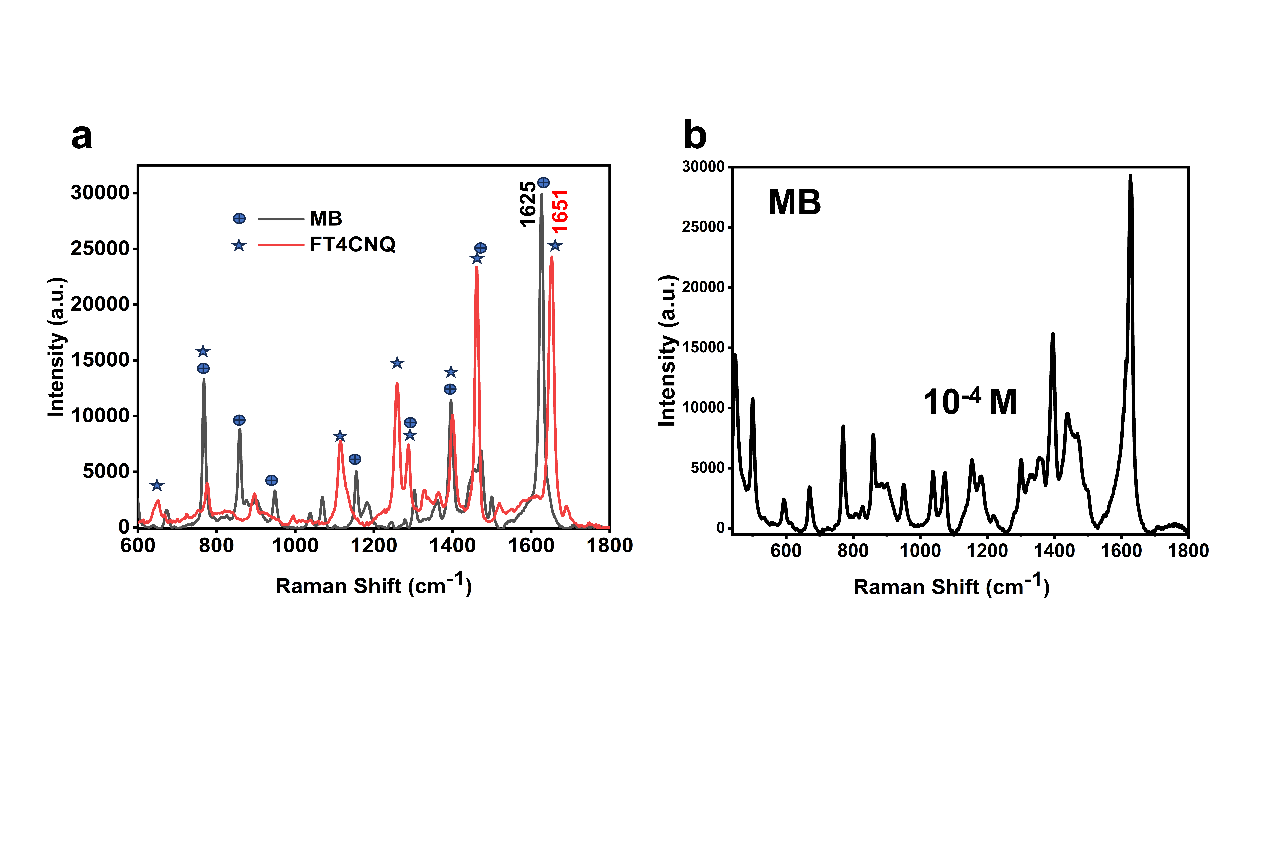


**Figure S18.** Raman spectra of (a) MB and F_4_TCNQ (b) MB on bare AAO substrate**.**

**Table S2.** A comparative analysis of limit of detection (LOD) and stability among different reported SERS substrates.

| Substrates  types  Plasmon free substrates  Noble metal substrates | samples | LOD R6G | LOD MB | Stability  (Days) | References |
| --- | --- | --- | --- | --- | --- |
|  | F_4_TCNQ/MoS_2_/AAO | 5$\times$10^-14^ | 5$\times$10^-16^ | 90 | This work |
|  | 1T’-WTe_2_ | 5$\times$10^-14^ | / | 12 | ^[3]^ |
|  | Few layer MoS_2_ | 1$\times$10^-9^ | / | / | ^[4]^ |
|  | 1T’MoS_2_ | 1$\times$10^-9^ | / | / | ^[5]^ |
|  | 1T’-MoSe_2_ | 1$\times$10^-8^ | / | / | ^[6]^ |
|  | 1T’-MoTe_2_ | 5$\times$10^-9^ | 1$\times$10-8 | 45 | ^[7]^ |
|  | 1T-ReS_2_ | 1$\times$10^-9^ | 1$\times$10^-9^ | / | ^[8]^ |
|  | NbTe | 1$\times$10^-7^ | 1$\times$10-9 | / | ^[9]^ |
|  | W18O49/MoS_2_ | 1$\times$10^-9^ | / | 75 | ^[10]^ |
|  | NbS_2_ |  | 5$\times$10^-14^ | 14 | ^[11]^ |
|  | MoS_2_/Graphene | 5$\times$10^-12^ | / | / | ^[12]^ |
|  | PdS_2_ | 5$\times$10^-9^ | / | 30 | ^[13]^ |
|  | Pt/MoS_2_/TiO_2_ | 1$\times$10^-9^ | / | / | ^[14]^ |
|  | DFP-4T | / | 5$\times$10-9 | / | ^[15]^ |
|  | Au/h-BN/Ag | 1$\times$10^-12^ |  | 80 | ^[16]^ |
|  | Au@Ag/Si | 1$\times$10^-8^ |  | 21 | ^[17]^ |
|  | Ag/MoS_2_ | 1$\times$10^-12^ |  | / | ^[18]^ |

**
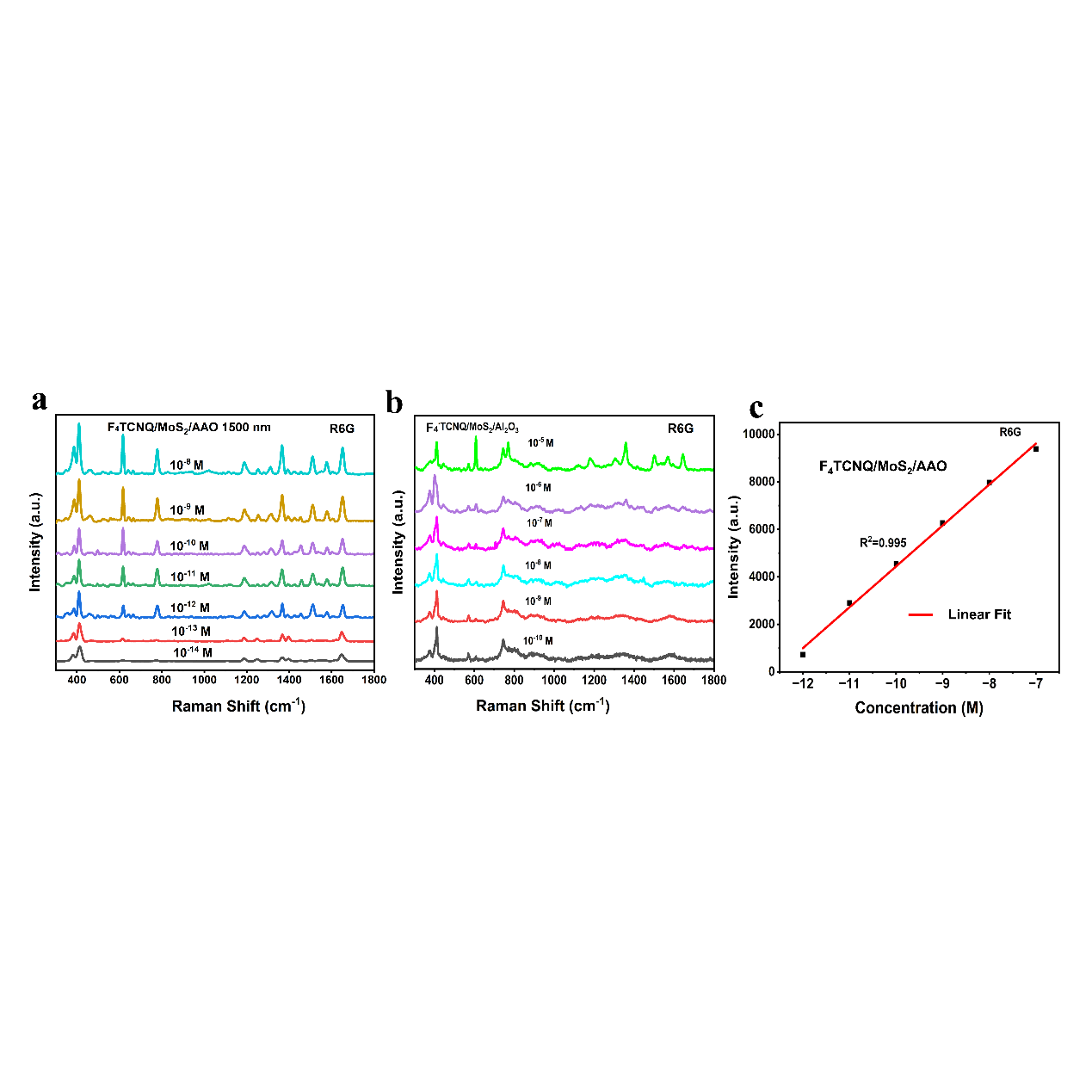
**

**Figure S19.** SERS spectra of R6G on different substrates. a) Raman spectra of R6G on a) F_4_TCNQ/MoS_2_/AAO (1500nm). b) F_4_TCNQ/MoS_2_/Al_2_O_3_. c) Co-relation factor of R6G at 613 cm^-1^.

Among the different substrates, AAO (1500 nm) exhibits the most robust Raman signals for R6G displaying a LOD of 10^-14^ M depicted in Figure S19a. This highlights the effective enhancement of SERS performance through optical resonance in the nanopores, molecular resonance, and CT mechanism. Compared to AAO nanotemplates, the SERS mechanism of R6G on flat Al_2_O_3_ is shown in Figure S19b showing a LOD of 10^-10^ M. The Raman vibrational modes of R6G molecules are slightly different from those reported substrates presented in Table S3, which may be attributed to structural distortion caused by CT and molecular polarization induced by varying degrees of chemisorption on the samples. The high value of the correlation coefficient (R^2^) between the intensity of the 613 cm^-1^ peak and the concentration of R6G demonstrates a strong linear relationship between the SERS intensity and the concentration of the analyte, leading to good reliability, as depicted in Figure S19c.

**Figure S20.** a) Reproducibility of MB on F_4_TCNQ/MoS_2_/AAO. b) SERS mapping of MB.

**Table S3.** Raman characteristic peaks of R6G.^[19,20]^

| Raman Shift (cm^-1^) | Band assignment |
| --- | --- |
| 614 | C-C-C ring in-plane bend |
| 776 | C-H out-of-plane bend |
| 1131 | C-H in-plane bend |
| 1185 | C-C stretching vibration bend |
| 1312 | Aromatic C-C stretching |
| 1364 | Aromatic C-C stretching |
| 1419 | Aromatic C-C stretching/C-H vibration |
| 1506 | Aromatic C-C stretching |
| 1575 | Aromatic C-C stretching |
| 1601 | Aromatic C-C stretching |
| 1648 | Aromatic C-C stretching/C-H vibration |

**Table S4.** Comparison of photodegradation of SERS substrate.

| SERS Substrate | Target molecule | Degradation time | References |
| --- | --- | --- | --- |
| F_4_TCNQ/MoS_2_/AAO  F_4_TCNQ/MoS_2_/Al_2_O_3_ | MB  MB | 80 min  60 min | This work |
| P_3_HT@Ag_2_NCN | MB | 8h | ^[21]^ |
| Ag@ZnO@Bi_2_WO_6_ | R6G | 140 min | ^[22]^ |
| Ag/TiO_2_ cotton fabric | PATP | 180 min | ^[23]^ |
| TiO_2_/Ag NPs | R6G | 120 min | ^[24]^ |
| Ag NPs/TiO_2_ nanotube arrays | R6G | 150 min | ^[25]^ |
| TiO_2_@PLDOPA@Ag NPs | MB | 5 h | ^[26]^ |
| MoO_2_/ZnSe | MB | 180 min | ^[27]^ |
| Ag-decorated ZnSe | R6G | 160 min | ^[28]^ |

**References**

[1] Q. Wei, Y. Fu, G. Zhang, D. Yang, G. Meng, S. Sun, *Nano Energy* **2019**, *55*, 234.

[2] C. Gu, S. Man, J. Tang, Q. Ye, Z. Yu, *IOP Conf Ser Mater Sci Eng* **2018**, *394*, 022049.

[3] L. Tao, K. Chen, Z. Chen, C. Cong, C. Qiu, J. Chen, X. Wang, H. Chen, T. Yu, W. Xie, S. Deng, J.-B. Xu, *J Am Chem Soc* **2018**, *140*, 8696.

[4] B. P. Majee, S. Mishra, R. K. Pandey, R. Prakash, A. K. Mishra, *The Journal of Physical Chemistry C* **2019**, *123*, 18071.

[5] E. Er, H. L. Hou, A. Criado, J. Langer, M. Möller, N. Erk, L. M. Liz-Marzán, M. Prato, *Chemistry of Materials* **2019**, *31*, 5725.

[6] Y. Yin, P. Miao, Y. Zhang, J. Han, X. Zhang, Y. Gong, L. Gu, C. Xu, T. Yao, P. Xu, Y. Wang, B. Song, S. Jin, *Adv Funct Mater* **2017**, *27,* 1606694.

[7] J. P. Fraser, P. Postnikov, E. Miliutina, Z. Kolska, R. Valiev, V. Švorčík, O. Lyutakov, A. Y. Ganin, O. Guselnikova, *ACS Appl Mater Interfaces* **2020**, *12*, 47774.

[8] P. Miao, J. Qin, Y. Shen, H. Su, J. Dai, B. Song, Y. Du, M. Sun, W. Zhang, H. Wang, C. Xu, P. Xu, *Small* **2018**, *14,* 1704079.

[9] K. Wang, Z. Guo, Y. Li, Y. Guo, H. Liu, W. Zhang, Z. Zou, Y. Zhang, Z. Liu, *ACS Appl Nano Mater* **2020**, *3,* 11363.

[10] M. Li, X. Fan, Y. Gao, T. Qiu, *J Phys Chem Lett* **2019**, *10*, 4038.

[11] X. Song, Y. Wang, F. Zhao, Q. Li, H. Q. Ta, M. H. Rümmeli, C. G. Tully, Z. Li, W.-J. Yin, L. Yang, K.-B. Lee, J. Yang, I. Bozkurt, S. Liu, W. Zhang, M. Chhowalla, *ACS Nano* **2019**, *13*, 8312.

[12] S. A. Ghopry, M. A. Alamri, R. Goul, R. Sakidja, J. Z. Wu, *Adv Opt Mater* **2019**, *7*, 1801249.

[13] Z. Lei, X. Zhang, Y. Zhao, A. Wei, L. Tao, Y. Yang, Z. Zheng, L. Tao, P. Yu, J. Li, *Nanoscale* **2022**, *14*, 4181.

[14] J. Dong, J. Huang, A. Wang, G. V. Biesold-McGee, X. Zhang, S. Gao, S. Wang, Y. Lai, Z. Lin, *Nano Energy* **2020**, *71*, 104579.

[15] G. Demirel, R. L. M. Gieseking, R. Ozdemir, S. Kahmann, M. A. Loi, G. C. Schatz, A. Facchetti, H. Usta, Nat. Commun. **2019**, 10, 5502.

[16] N.-Y. Kim, Y.-C. Leem, S.-H. Hong, J.-H. Park, S.-Y. Yim, *ACS Appl Mater Interfaces* **2019**, *11*, 6363.

[17] C. Zhang, S. Z. Jiang, C. Yang, C. H. Li, Y. Y. Huo, X. Y. Liu, A. H. Liu, Q. Wei, S. S. Gao, X. G. Gao, B. Y. Man, *Sci Rep* **2016**, *6*, 25243.

[18] J. Li, H. Liu, S. Chen, X. Liang, Y. Gao, X. Zhao, Z. Li, C. Zhang, F. Lei, J. Yu, *J Phys Chem Lett* **2022**, *13*, 5815.

[19] P. Hildebrandt, M. Stockburger, *J Phys Chem* **1984**, *88*, 5935.

[20] F. Liao, L. Cheng, J. Li, M. Shao, Z. Wang, S.-T. Lee, *J Mater Chem C Mater* **2013**, *1*, 1628.

[21] L. Xu, T. Wang, X. Li, Z. Chen, *Chemosensors 2022, Vol. 10, Page 469* **2022**, *10*, 469.

[22] I. Korkmaz, M. Sakir, G. Sarp, S. Salem, I. Torun, D. Volodkin, E. Yavuz, M. S. Onses, E. Yilmaz, *J Mol Struct* **2021**, *1223*, 129258.

[23] F. Ge, Y. Chen, A. Liu, S. Guang, Z. Cai, *Cellulose* **2019**, *26*, 2689.

[24] H. Y. Wu, H. C. Lin, Y. H. Liu, K. L. Chen, Y. H. Wang, Y. S. Sun, J. C. Hsu, *Molecules 2022, Vol. 27, Page 6755* **2022**, *27*, 6755.

[25] H. Zhai, C. Zhu, X. Wang, Y. Yuan, H. Tang, *Front Chem* **2022**, *10*, 992236.

[26] H. Mazlumoglu, M. Yilmaz, Phys. Chem. Chem. Phys. **2021**, 23, 13396.

[27] M. Liu, X. Hu, C. Zhang, M. Shafi, L. Ma, B. Lv, A. Rahim, M. Saleem, L. Zhao, *Sens Actuators B Chem* **2024**, *398*, 134688.

[28] M. Shafi, P. Duan, W. Liu, W. Zhang, C. Zhang, X. Hu, C. Liu, S. Wali, S. Jiang, C. Zhang, B. Man, M. Liu, *Sens Actuators B Chem* **2023**, 133410.
